# The role of colony morphotype in shaping gene essentiality in *Mycobacteroides abscessus*

**DOI:** 10.1073/pnas.2500719122

**Published:** 2025-09-30

**Authors:** Brittany N. Ross, Emma Evans, Frances Diggle, Paul Briaud, Marvin Whiteley

**Affiliations:** ^a^Center for Microbial Dynamics and Infection, School of Biological Sciences, Georgia Institute of Technology, Atlanta, GA 30332; ^b^CF@LANTA Emory-Children’s Cystic Fibrosis Center, Atlanta, GA 30322; ^c^Department of Biology, Georgia State University, Atlanta, GA 30303

**Keywords:** *Mycobacteroides abscessus*, cystic fibrosis, morphotype, Tn-seq, essential genes

## Abstract

Changes in bacterial colony morphology are common during chronic human infections and are thought to provide a survival advantage. In the human pathogen *Mycobacteroides abscessus* (MAB), a unidirectional transition from a smooth (MAB^S^) to rough (MAB^R^) morphotype frequently occurs during chronic infection. This transition has profound clinical implications, as MAB^R^ induces a heightened proinflammatory response, contributing to increased morbidity. To better understand this phenomenon, we used transposon insertion site sequencing (Tn-seq) to identify genes essential for the survival of MAB^S^ and MAB^R^. Our analysis revealed distinct genetic requirements for growth in vitro, including several genes involved in responding to environmental stresses. Notably, some of these uniquely essential genes are therapeutic targets in mycobacteria. In a murine infection model, the divergence in essential gene profiles between MAB^S^ and MAB^R^ was even more pronounced, driven partly by the differing host immune responses elicited by each morphotype. These findings demonstrate that the transition from a smooth to rough morphotype not only impacts MAB’s survival strategies but also highlights the importance of considering morphotype-specific genetic and functional adaptations when developing therapeutic approaches. Our work underscores the critical need to incorporate morphotype conversion into the prioritization of drug targets, as targeting morphotype-specific vulnerabilities could improve treatment outcomes for infections caused by this pathogen.

## Significance

Bacteria often adapt to chronic human infection by altering their outer surface, leading to changes in appearance (morphotype) on agar plates. These morphotypic changes often worsen outcomes by eliciting different host responses. While noted in various bacteria, their impact on genes essential for growth and survival remains unclear. We addressed this by defining the essential genes of smooth and rough morphotypes of the human pathogen *Mycobacteroides abscessus* (MAB). MAB often transitions to the rough morphotype during chronic infection, increasing morbidity. Our findings revealed unique essential genes in MAB morphotypes both in vitro and in a murine model, including some prioritized as therapeutic targets. These results highlight the importance of defining morphotype-specific essential genes in developing therapies.

When microbes chronically colonize the human body, they often undergo adaptations that enhance their fitness. The outer surface of a microbe is the primary interface with the host, and mutations affecting the chemical composition of the outer surface have been observed in many bacteria during human chronic infection. These mutations are frequently linked to changes in the microbe’s appearance when cultured on agar plates, referred to as the colony morphotype. Changes in morphotype have been associated with changes in infection chronicity (1, 2), pathogenicity (3, 4), and antibiotic susceptibility (5, 6).

*Mycobacteroides abscessus* (MAB, formerly known as *Mycobacterium abscessus*) is a fast-growing nontuberculous mycobacterium (NTM). MAB can cause a wide array of human infections, the most well-studied being chronic pulmonary infections in individuals with underlying lung disease, such as cystic fibrosis (CF) (7). More recently MAB has been identified as a cause of skin, soft tissue, or disseminated infections following surgery (8–14). MAB is found in water samples sourced from premise plumbing including sinks and shower heads (15–18), which is suspected to be the main source of nosocomial infection.

During chronic human infection, MAB often transitions from a smooth shiny colony (MAB^S^) morphotype to a rough colony with an irregular surface (MAB^R^), and this adaptation leads to worse prognosis for the infected individual (19, 20). This morphotype transition is caused by mutation(s) in genes encoding for proteins involved in biosynthesis or transport of surface-associated glycopeptidolipids (GPLs), which are a group of lipopeptides decorated with O-methylated and O-acetylated deoxy-hexoses. The lack of GPLs on the outer surface leads to the rough colony morphology of MAB^R^. MAB^R^ strains have been shown to be more virulent in a zebrafish infection model (21) and immunocompromised murine respiratory models (22–24), and the transition to MAB^R^ is correlated with lung function decline in people with CF (19, 20). Conversely, MAB^S^ produces more robust in vitro biofilms and has sliding motility (25). Interestingly, morphotype conversion to MAB^R^ during chronic human infection mimics the evolutionary trajectory of *Mycobacterium tuberculosis* (Mtb), which exhibits a rough morphotype and arose from the smooth morphotype soil bacterium, *Mycobacterium canettii* (26, 27). Like Mtb, MAB globally distributed dominant circulating clones (DCCs) have emerged with increased pathogenicity (28) and gene acquisition indicates beneficial evolution toward human infectivity (29, 30).

Although phenotypic differences between MAB^R^ and MAB^S^ have been attributed to the presence/absence of GPLs, it is well known that mutations that impact colony morphotype in other bacteria can impact numerous, seemingly unconnected, pathways. For example, *Pseudomonas aeruginosa* has been shown to adapt to CF lung infection by changing to a mucoid morphotype that overproduces the exopolysaccharide alginate. The mucoid morphotype results in complex physiological changes not directly attributed to the presence/absence of the alginate capsule, including increased release of hydrogen cyanide (31, 32). *Vibrio cholerae* can also exist as a rough or a smooth colony morphotype that exhibit differences in acetate metabolism, gluconeogenesis, and chemotaxis (33). Thus, morphological changes often result in physiological changes that are not directly attributable to the presence/absence of an extracellular component.

Given the increased pathogenesis of MAB^R^, it is critical to understand how morphotype switching impacts its physiology and pathogenesis. To accomplish this, we employed transposon insertion site sequencing (Tn-seq). Tn-seq is a powerful technique which combines high-throughput transposon mutant screening with next-generation sequencing, to identify genes that are essential for fitness in an environment of interest. Previous studies have used Tn-seq in MAB to identify phylogenic-cluster specific essential genes (34), genes critical for survival in host cells (35, 36), and mechanisms of antibiotic resistance (37–39). Here, we used Tn-seq to identify the essential genes of MAB^S^ and MAB^R^, both in vitro and in a murine model of infection. We found that the two MAB morphotypes have unique gene requirements both in vitro and in vivo, and some of the differentially essential genes in vivo are due to the altered immune response to MAB^R^. We also show that the presumed MAB essential genes *pknA* and *sdh2*, which have been prioritized for therapeutic development in MAB and related mycobacterial species (40–42), are only essential in a single morphotype in vitro.

## Results

### Construction and Analysis of MAB^S^ and MAB^R^ Mutant Libraries.

The model MAB strain ATCC 19977, first isolated from an abscess in 1953, was used in these studies(43). MAB ATCC 19977 is a member of the dominant circulating clonal 1 complex (DCC1), which are linked to 20% of MAB infections (44). Upon acquisition from ATCC, ATCC 19977 appeared as both MAB^S^ and MAB^R^ morphotypes on agar plates. Single colonies of each morphotype were isolated (7), and whole genome sequencing performed. Genome comparison revealed MAB^R^ contained a single base pair deletion in MAB_4098c (nucleotide position 6,759 of the gene), which resulted in a frameshift mutation. MAB_4098c encodes Mps2, a nonribosomal peptide synthetase required for GPL synthesis (*SI Appendix*, Table S1). Mutations in the *mps1-mps2* cluster have previously been shown to lead to the MAB^R^ morphotype (45, 46).

Transposon libraries were generated in the MAB^S^ and MAB^R^ ATCC 19977 strains using the MycoMar bacteriophage containing the HiMar1 Mariner transposon (47). This transposon inserts randomly into TA dinucleotides. Of the 91,449 possible TA insertion sites in the ATCC 19977 genome, 41,130 and 54,603 sites were occupied by the transposon in the MAB^S^ and MAB^R^ transposon libraries, respectively (*SI Appendix*, Table S2). On average, this equates to an insertion every 93 to 123 bp in the ATCC 19977 genome with 8 to 11 distinct Tn insertions present in an average 1 kb nonessential MAB gene.

**Fig. 1.**
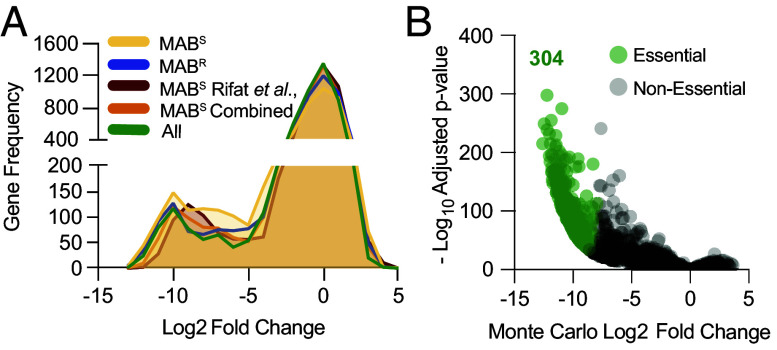
Identification of MAB essential genes. (*A*) Frequency plot of the log_2_-transformed difference between observed and fitness-neutral pseudodata for each gene in the three Tn-seq datasets used in this study. “MAB^S^” and “MAB^R^” datasets were generated in this study and “MAB^S^ Rifat” was previously generated using a MAB^S^ strain (55). “All” shows an analysis of all datasets combined. (*B*) Identification of conserved essentials genes using all the datasets combined. Fold-change in transposon mutant abundance from the observed and fitness-neutral pseudodata of each gene is shown along with −log_10_ adjusted *P*-values. We defined the statistical parameters for classification of a gene as essential using 22 genes universally essential across bacteria (54). The 304 genes that meet these statistical parameters for essentiality (log_2_ fold-change ≤ −8, adjusted *P*-value ≤ 3 × 10^−34^) are shown in green.

To define the essential genes in both MAB morphotypes, we used a computational pipeline that identifies essential genes by comparing observed transposon mutants in a mutant library to in silico generated transposon frequencies (referred to as pseudodata) under a null model where no gene is essential, the latter calculated using a Monte Carlo approach (48–53). For this analysis, we used data from 22 universally essential bacterial genes (54) to define the statistical parameters for classification of a gene as essential (log_2_ fold-change ≤ −8, adjusted *P*-value ≤ 3 × 10^−34^; Dataset S1*A*). In addition to our MAB^S^ and MAB^R^ transposon libraries, we also performed this analysis on a publicly available Tn-seq dataset from MAB^S^ ATCC 19977 [Rifat et al. (55), *SI Appendix*, Table S2]. The three Tn-seq datasets showed similar essential genes (Fig. 1*A*). Analysis of all three datasets combined identified 304 of the 4,992 coding genes as essential (Fig. 1*B* and Dataset S1*B*), and 297 of these 304 genes (98%) are core genes based on a pangenome analysis of 34 globally representative genomes (*SI Appendix*, Fig. S1 and Datasets S1 and S2). Analyzing data generated only in this study identified 85.8% of essential genes found using all three studies (*SI Appendix*, Fig. S2 and Dataset S3). Small genes or genes with few transposon insertion sites are more prone to being incorrectly classified as essential. Monte Carlo analysis excluded genes with limited insertion sites from being deemed essential due to low confidence. Here, 6 genes smaller than 300 bp and 9 genes with as few as four TA sites, were found to be essential (*P*-value ≤ −6.97 × 10^−35^; Dataset S1*B* and *SI Appendix*, Fig. S3).

### MAB Morphotypes have Unique Gene Requirements.

An analysis of the essential genes in MAB^S^ and MAB^R^ revealed 79 unique essential genes in MAB^S^ and 65 unique essential genes in MAB^R^ (Dataset S4 *A* and *B*). As we used highly stringent criteria for classifying essential genes (log_2_ fold-change ≤ −7, adjusted *P*-value ≤ 2 × 10^−14^; Dataset S4 *A* and *B*), it is possible that one gene may satisfy the criteria for essentiality in one morphotype but fall just short of the criteria for the other. As our goal is to focus on genes that are essential in each morphotype but have minimal impact on fitness in the other, we performed two additional analyses to prioritize genes of interest:

#### Differential transposon frequency analysis.

**Fig. 2.**
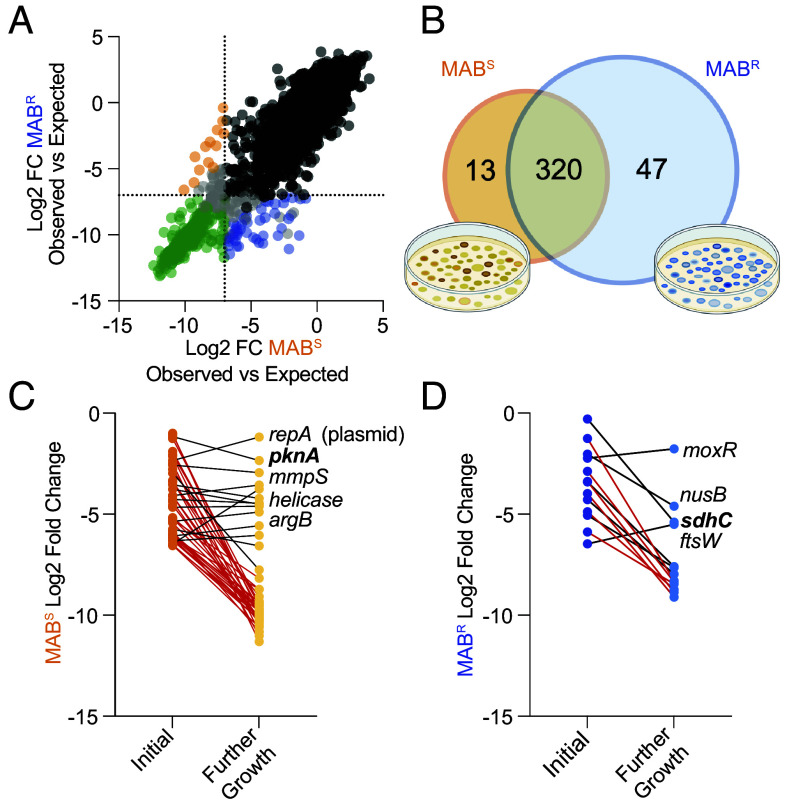
MAB morphotypes have unique essential genes. (*A*) MAB^S^ and MAB^R^ essential genes identified using Monte Carlo and differential transposon frequency analysis. (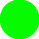) are genes essential in both morphotypes, (●) are genes not essential in either morphotype, (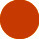) are genes essential in MAB^S^, and (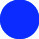) are genes essential in MAB^R^. (*B*) Venn diagram of the MAB^S^ and MAB^R^ essential genes revealed 13 and 47 unique essential genes. (*C*) Monte Carlo log_2_ fold-change for the 47 MAB^S^ essential genes after further in vitro growth. “Initial” refers to data from the original transposon populations and ‘Further growth’ is data after additional in vitro growth. Red lines (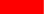) indicate a significant fitness defect upon growth while black lines (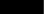) indicate no defect in fitness. (*D*) Identical analysis to that in panel *C* but using the 13 MAB^R^ essential genes.

We compared the frequencies of transposon mutants in the MAB^S^ and MAB^R^ transposon libraries (Fig. 2*A* and *SI Appendix*, Fig. S4 and Dataset S4*B*), prioritizing genes that showed greater than 4-fold-change in frequency between the morphotypes (*P* < 0.05). This reduced the number of unique essential genes to 13 in MAB^S^ and 47 in MAB^R^ (Fig. 2*B*). Of the MAB^S^ essential genes that are neighbored by another essential gene, four are downstream of genes essential in both morphotypes (*SI Appendix*, Fig. S5). Among the 47 MAB^R^ essential genes, nine are neighbored by another MAB^R^ essential gene and three are downstream of a gene essential in both morphotypes (*SI Appendix*, Fig. S5). The remaining morphotype-specific essential genes are either in an operon with nonessential genes or are not part of an operon. While morphotype essential genes may result from polar effects, the data suggest that polar effects of transposon insertion are likely not a primary driver of essentiality of these genes.

#### Differential fitness analysis of mutants after further growth.

**Fig. 3.**
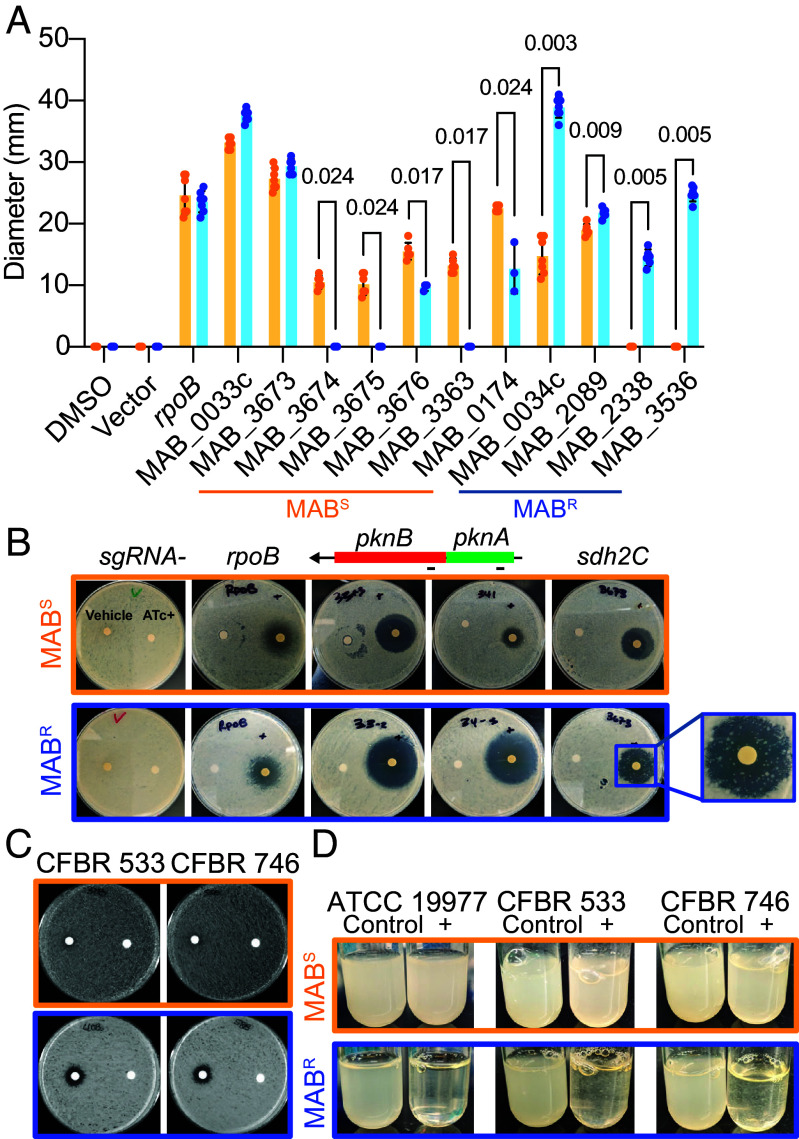
Confirmation of morphotype-specific gene essentiality using CRISPRi. (*A*) Diameter of zones of inhibition quantified using disc diffusion assays for MAB strains carrying each CRISPRi construct. For these experiments, filter discs containing the sgRNA inducer ATc were placed on lawns of MAB strains carrying the CRISPRi constructs. The diameter of the zone of inhibition for each morphotype were compared for each CRISPRi construct. As controls, the vector only control (no sgRNA), two genes essential in both morphotypes (*rpoB* and *pknB*) and DMSO (solvent for ATc) are shown. Displayed *P*-values were calculated by the Mann–Whitney test. (*B*) Images of the zone of growth inhibition for select genes in both morphotypes. Note, the size of the inhibition zone is dictated by many factors including the efficiency of each sgRNA. Thus, inhibition zone size can only be compared between morphotypes, not between genes. A zoomed image is inset for *sdh2C* + CRISPRi in MAB^R^ which repeatably showed colonies growing in the zone of inhibition. (*C* and *D*) To assess the morphotype-specific essentiality in CF clinical isolates, CRISPRi targeting *pknA* was done in two pairs of MAB^S^ and MAB^R^ obtained from two people with CF (CFBR 533 and CFBR 746). Disc diffusion assays (*C* and *D*) *pknA* repression during planktonic growth are shown. Representative images are shown after 96 h of growth.

Fitness defects of transposon mutants in Tn-seq libraries is impacted by the number of cell divisions that the population is allowed to undergo. Thus, as our approach defines essentiality by the frequency of transposon mutants compared to a null model, some transposon mutants may not be deemed essential in the initial transposon library but might be upon further growth. To identify such genes, the MAB^S^ and MAB^R^ transposon libraries were grown for an additional five cell divisions and essential genes identified (Dataset S5 *A* and *B*), and no difference in growth rate was observed (*SI Appendix*, Fig. S6). Of the 47 MAB^R^ essential genes prioritized after differential transposon frequency analysis (Fig. 2*B*), 33 genes have a fitness defect MAB^S^ after additional growth and 14 do not (black lines, Fig. 3*C*). Of the 13 MAB^S^ essential genes prioritized after differential transposon frequency analysis (Fig. 2*B*), 7 did not show a significant fitness defect after additional growth in MAB^R^ (black lines, Fig. 2*D*). We focused on these 21 genes (14 MAB^R^ and 7 MAB^S^) for validation as they are essential in one morphotype and have limited impact on fitness in the other morphotype.

### Validation of Unique Essential Genes.

To begin to validate our Tn-seq results, we used clustered regularly interspaced short palindromic repeat interference (CRISPRi) to test the essentiality of a subset of the 21 morphotype-specific genes. Single guide RNAs (sgRNAs) targeting genes of interest were constructed on a plasmid under control of a tetracycline-inducible promoter. Induction of these sgRNAs and a nuclease inactive Cas9 with anhydrotetracycline (ATc) reduces the target mRNA, and the impact on MAB growth can be assessed.

As controls, we induced Cas9 in a MAB strain lacking an sgRNA (negative control) or a strain containing an sgRNA targeting *rpoB* (MAB_3869c), which encodes for the beta subunit of RNA polymerase and is essential in both MAB^S^ and MAB^R^ (positive control). Induction of Cas9 in a strain lacking an sgRNA had no effect on growth (Fig. 3*A*). However, induction of the sgRNA targeting *rpoB* eliminated growth in both morphotypes at high ATc concentrations (i.e., a zone of clearing was observed around the ATc disc), confirming that *rpoB* is essential and validating our CRISPRi approach (Fig. 3 *A* and *B*). Six MAB^S^ and four MAB^R^ specific essential genes were targeted with CRISPRi, and all but one, MAB_3673, recapitulated the Tn-seq results (Fig. 3*A*). MAB_3673 encodes succinate dehydrogenase 2 subunit C (*sdh2C*), which is essential in MAB^S^ but not MAB^R^. Induction of the guide RNA targeting *sdh2C* results in substantial growth inhibition of both MAB^S^ and MAB^R^ (Fig. 3*B*). However, a large number of colonies were observed growing within the MAB^R^ zone of clearing (Fig. 3*B*), which is likely why our Tn-seq data did not reveal this gene as essential in MAB^R^. Three colonies were isolated from the zone of clearing and restreaked onto media with and without ATc. All three colonies grew in the presence and absence of ATc, indicating that growth in the presence of the guide RNA inducer was heritable (*SI Appendix*, Fig. S7). To ensure that this heritability was not due to mutations in the CRISPRi plasmid, we performed sequencing of the strains. No mutations were identified in the Cas9 or sgRNA genes (*SI Appendix*, Table S3) (56). However, mutations were identified in the MAB genome (*SI Appendix*, Table S3) suggesting that strains growing within the zone of clearing in the presence of ATc likely acquired suppressor mutations on the MAB^R^ genome, explaining the difference in *sdh2C* mutant frequency between the morphotypes observed in the Tn-seq data.

We next focused on the serine/threonine protein kinases A and B (*pknA* is MAB_0034c and *pknB* is MAB_0033c*)*, which are essential in *M. tuberculosis* (57). CRISPRi confirmed our Tn-seq data that both morphotypes require *pknB,* while *pknA* is essential in MAB^R^, but not MAB^S^ (Fig. 3 *A* and *B* and *SI Appendix*, Fig. S8). The morphotype-specific essentiality of *pknA* was not due to differential efficiency or polar effects of the CRIPSRi system used, as qRT-PCR showed no difference in abundance of the transcripts in the two morphotypes (*SI Appendix*, Fig. S8). To determine whether the MAB^R^-specific essentiality of *pknA* is specific to MAB ATCC 19977, we selected paired CF clinical MAB^S^ and MAB^R^ strains that had been isolated from two people with CF (7). Instead of *mps2* mutations like we found in ATCC 19977, the clinical isolates EN40 and EN58 from CFBR553 and CFBR746, respectively, have a mutation in *mps1,* which eliminates GPL and causes the MAB^S^-to-MAB^R^ transition (7). CRISPRi targeting of *pknA* revealed a zone of clearing (Fig. 3*C*) and reduced growth (Fig. 3*D*) in MAB^R^, but not MAB^S^, indicating that the importance of *pknA* for MAB^R^ fitness is not restricted to the ATCC 19977 strain.

### Both MAB Morphotypes can Establish a Cutaneous Abscess Infection.

**Fig. 4.**
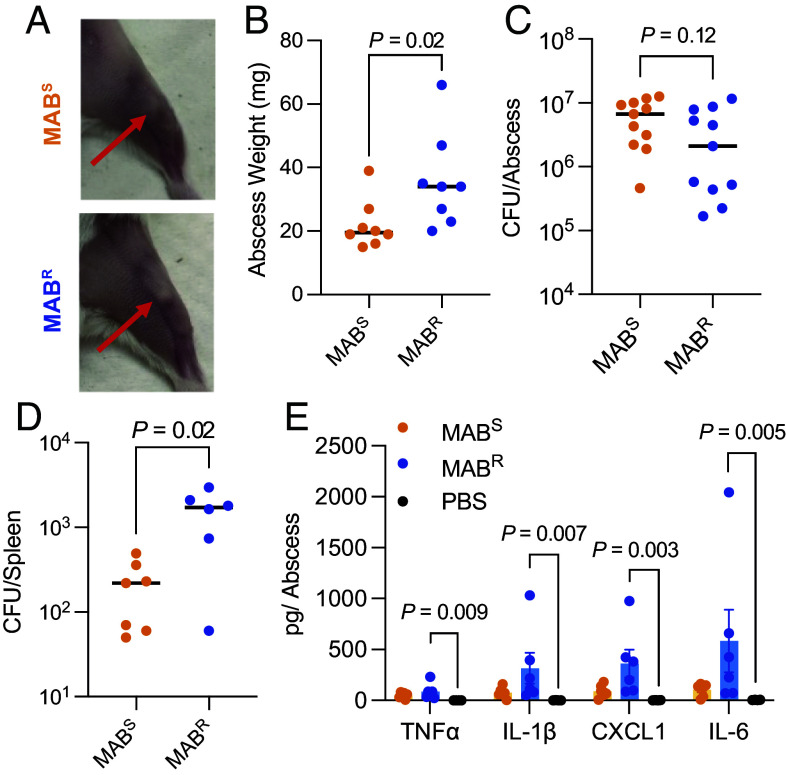
Development of a MAB abscess model. (*A*) Image of abscesses 3 d postinfection with MAB morphotypes. 10^7^ CFU was injected into the inner thighs of Swiss Webster mice to induce the infection. (*B*) Weight of abscesses 3 d postinfection. (*C*) Bacterial colony-forming units (CFUs) in the abscess and (*D*) spleen 3 d postinfection. (*E*) Cytokine and chemokine levels in the MAB-infected abscesses compared to a PBS control. CFU comparisons were analyzed using the Mann–Whitney. Cytokine levels were compared using the Kruskal–Wallis test.

We next asked whether MAB^S^ and MAB^R^ have unique essential genes in an infection model. While in vivo MAB models have primarily focused on respiratory infections, cutaneous infections, such as abscesses, are a common manifestation and are increasingly observed (8–13, 16, 58–65). Here, we developed a murine abscess model for studying MAB infection. In this model, 10^7^ MAB^S^ or MAB^R^ are injected into the inner thigh of Swiss-Webster mice, and the resulting abscess collected after 72 h (Fig. 4*A*). At this timepoint, MAB^R^ infected abscesses weighed more (Fig. 4*B*), but the bacterial burden did not differ significantly between the morphotypes (Fig. 4*C*). However, MAB^R^ led to significantly higher dissemination to the spleen (Fig. 4*D*). Without GPL, MAB^R^ has exposed proinflammatory antigens on its surface, which in other murine infection models has been shown to activate Toll-like receptor-2 (TLR-2) and increases tumor necrosis factor-alpha (TNF-α)-mediated inflammation (66, 67). To test whether this is occurring in the abscess model, we performed cytokine and chemokine analyses on abscess homogenates (Fig. 4*E*). Compared to PBS controls, MAB^R^-infected abscesses exhibited elevated proinflammatory markers, including TNF-α, IL-1β, IL-6, and the neutrophil chemoattractant CXCL1 (Fig. 4*E*). These data indicate that as in other infection models (22–24, 67–72), MAB^R^ induces a proinflammatory response in the abscess infection model.

### Morphotypes Require Unique Essential Genes During Abscess Infection.

To identify genes required for establishing an abscess infection, we inoculated mice with MAB^S^ or MAB^R^ transposon libraries, collected abscesses 3 d postinfection, and determined transposon mutant frequencies. We chose 3 d postinfection because the morphotypes have similar bacterial burdens at this timepoint, and we found the Tn-seq samples cluster according to morphotype (*SI Appendix*, Fig. S9).

**Fig. 5.**
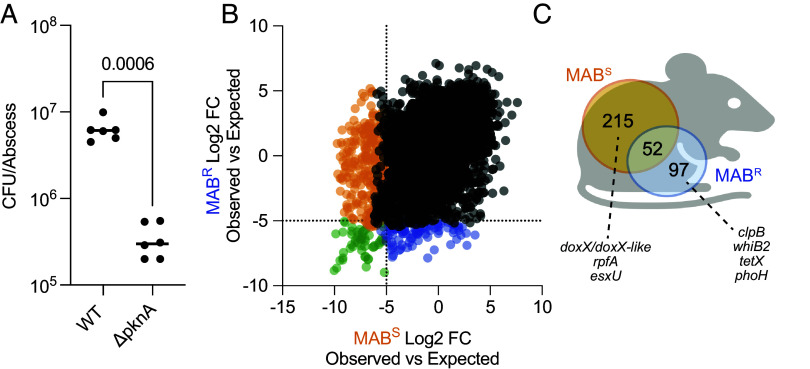
MAB essential genes in the abscess infection model. (*A*) While not essential in vitro in MAB^S^, *pknA* is critical for fitness in the abscess model. Data are bacterial CFUs in the abscess for WT MAB^S^ and the isogenic *pknA* deletion mutant (Δ*pknA*) 3 d postinfection. (*B*) Identification of morphotype-specific essential genes in the abscess infection model. Shown are the log_2_-transformed difference between observed and fitness-neutral pseudodata for each gene (circles) for both morphotypes. (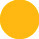) are MAB^S^-specific essential genes, (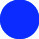) are MAB^R^-specific essential genes, and (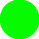) are shared essential genes. (*C*) Venn diagram of essential genes in the abscess in MAB^S^ and MAB^R^. The CFU comparison was done with the Mann-Whitney test.

We first asked whether the MAB^S^ and MAB^R^ unique in vitro essential genes are also important for fitness in the abscess model. Of the seven MAB^S^ unique in vitro essential genes, mutation in all of these genes negatively impacted MAB^R^ fitness during abscess infection (Dataset S6 *A* and *B*). Of the 14 unique in vitro essential genes in MAB^R^, only two, *ribD* and *argB*, did not have a fitness defect in MAB^S^ during abscess infection (Dataset S6 *A* and *B*). To further validate these in vivo findings, we evaluated the in vivo importance of *pknA*, which is essential in MAB^R^ but not MAB^S^ in vitro. For these experiments, we created an isogenic deletion mutant of *pknA* in MAB^S^ and assessed its fitness in the abscess. MAB^S^ *ΔpknA* exhibited an 18-fold reduction in CFUs compared to WT MAB^S^ in the abscess, confirming our Tn-seq data (28-fold reduction; Fig. 5*A*). These data reveal that most of the in vitro morphotype-specific essential genes have fitness defects in vivo, further supporting a role of the growth environment on conditionally essential genes.

We next asked whether there are new uniquely essential genes in MAB^S^ and MAB^R^ during abscess infection. For this analysis, we focused on the 4,083 nonessential in vitro genes that had mutants present in both the MAB^S^ and MAB^R^ transposon library. 215 and 97 essential genes were uniquely identified in MAB^S^ and MAB^R^ respectively, with 52 new genes deemed essential in both morphotypes (Fig. 5 *B* and *C*). Several genes uniquely essential to MAB^S^ are associated with establishing chronic infection including: the *doxX* gene (MAB_3707c and MAB_0705), which encodes a protein that together with SodA and SseA maintains cytosolic thiol homeostasis by mycothiol detoxification of reactive oxygen and nitrogen radicals in Mtb (73); MAB_0869c which encodes the resuscitation-promoting factor *rpfA* that orchestrates reactivation from dormancy (74); and MAB_3754c, which encodes *esxU* and is critical for intracellular survival (35). Interestingly, the *devSR* regulon (MAB_3890c-3891c) that encodes the 2-component sensor kinase responsible for sensing low oxygen and inducing dormancy provides a fitness advantage when mutated in MAB^S^ (Dataset S6*B*) (75, 76). MAB^S^ also requires genes important for recombination including *recR* (MAB_0320) and the putative Holliday junction repair protein (MAB_2883c), as well as genes important in sulfur metabolism (MAB_2178, MAB_1653, MAB_0406c, MAB_3129c). MAB^R^ requires several genes encoding proteins related to stress responses, including the genes encoding the *clpB* chaperone (MAB_4265c); redox-responsive regulator WhiB2 (MAB_1756); MAB_1496c encoding the secreted tetracycline-modifying protein TetX (77, 78); and MAB_1668, which encodes the putative phosphate starvation-inducible protein PhoH. MAB^R^ also requires genes that encode proteins that respond to DNA damage including RecF (MAB_0004) and MAB_3012, the latter of which encodes a probable methylated-DNA-protein-cysteine methyltransferase which is an adaptive response to DNA alkylation damage (79).

### Immune Activation During MAB^S^ Infection Leads to Convergence of Essential Genes.

**Fig. 6.**
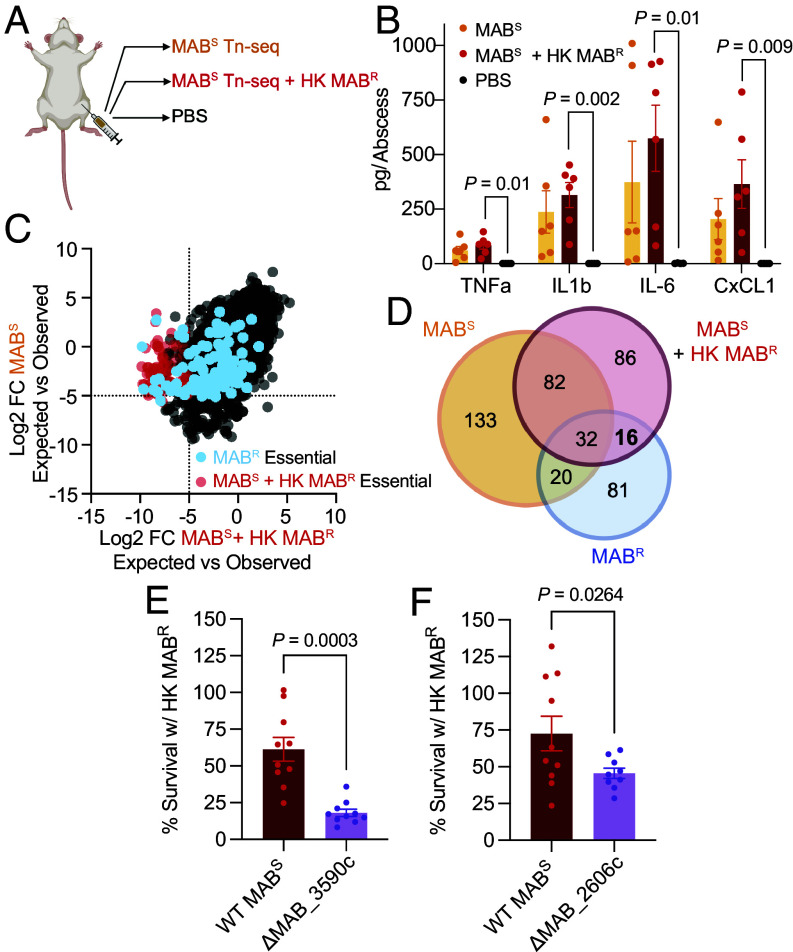
The differential immune response to MAB^S^ and MAB^R^ is responsible for a minority of MAB^S^-specific essential genes. (*A*) Graphical representation of the experimental design to assess whether MAB^S^-specific essential genes are a product of the differential immune response to MAB morphotypes. To expose MAB^S^ to a MAB^R^-like immune response in the abscess, the MAB^S^ transposon mutant library was infected with heat killed (HK) WT MAB^R^. (*B*) Cytokine and chemokine levels of abscesses infected with the MAB^S^ transposon library alone or with HK MAB^R^. Abscesses were collected 3 d postinfection and cytokine/chemokine levels compared to PBS-infected controls using the Kruskal–Wallis test. (*C*) Coinfection with HK MAB^R^ causes 16 MAB^R^-specific essential genes to become essential in MAB^S^. Shown are the log_2_-transformed difference between observed and fitness-neutral pseudodata for each gene (circles) for MAB^S^ and MAB^S^ coinfected with HK MAB^R^. (*D*) Venn diagram of essential genes in abscesses infected with MAB^S^, MAB^R^, or MAB^S^ + HK MAB^R^. (*E*&*F*) Percent survival of WT MAB and ΔMAB_3590c or ΔMAB_2606c mutants in the presence of HK MAB^R^ in the abscess.

It is well known that the host responds differently to infection by MAB^R^ and MAB^S^, with an increased proinflammatory response to MAB^R^ infection (22–24, 67–72). Thus, we hypothesized that this differential immune response is responsible for many of the unique morphotype-specific essential genes in the abscess. To test this hypothesis, we promoted a MAB^R^-like immune response in the presence of the MAB^S^ transposon mutant library, then assessed whether unique MAB^R^ essential genes became essential in MAB^S^. The primary proinflammatory antigens on the surface of MAB^R^ are PIM_2_ and lipoproteins, which activate Toll Like Receptor 2 (TLR2) (67). To mimic MAB^R^-mediated inflammation, heat-killed (HK) MAB^R^ or the TLR2 agonist PAM3CSK was coinoculated with the MAB^S^ transposon library into the abscess (Fig. 6*A*). Only abscesses coinoculated with HK MAB^R^ led to significant increases of IL-1β, TNFα, IL-6, and CXCL1 compared to PBS (Fig. 6*B*). In the presence of HK MAB^R^, an additional 108 genes became essential in MAB^S^. These genes included 16 of the 97 genes found uniquely essential in MAB^R^ during abscess infection (Fig. 6 *C* and *D* and Dataset S7 *A* and *B*). One of these genes (MAB_3590c) encodes MtrB, which encodes a sensor histidine kinase that controls cell division and is required for multidrug resistance in Mtb (80). Another gene that became essential with HK MAB^R^ is MAB_2606c, which has been shown to be upregulated in MAB upon exposure to hypoxic conditions (81). In addition, the gene encoding the *RidA* homolog (MAB_2910c) also became essential and encodes for 2-iminobutanoate/2-iminopropanoate deaminase and has been shown to protect against reactive imine intermediates (82). To validate these findings, deletion mutants of MAB_3590c and MAB_2606c were created in MAB^S^, and each mutant competed against WT MAB^S^ in the presence of HK MAB^R^ in the abscess model. As observed in the Tn-seq experiments, both deletion mutants showed a fitness defect compared to WT MAB^S^ (Fig. 6 *E* and *F*). These data indicate that induction of a MAB^R^-like immune response leads to 16 MAB^R^-unique essential genes becoming essential in MAB^S^ during abscess infection, indicating that the immune response to MAB^R^ is responsible for some, but not all, of the differences in morphotype-specific essential genes in the abscess.

## Discussion

During chronic human infection, MAB often transitions from a smooth to a rough morphotype. We found that this morphotype transition alters the genes required for fitness both in vitro and in vivo. The major difference between these morphotypes, and the simplest explanation for the change in gene requirements, is the loss of GPL in MAB^R^. The impact of GPL on morphotype specific gene essentiality is supported by work in other mycobacteria that do, and do not, produce GPL. For example, *sdh2C*, which is required in MAB^S^ but not MAB^R^, has been shown to be essential in GPL-positive *M. smegmatis* (83), but dispensable in GPL-lacking Mtb (84). In contrast *pknA*, which is required in vitro in MAB^R^ but not MAB^S^, is essential in both Mtb and *M. smegmatis* (85). This suggests that essentiality of some genes is correlated with the presence of GPL. The fact that GPL is localized to the outside of mycobacteria leads us to speculate that its presence impacts how MAB senses and responds to environmental cues. MAB^R^ shows increased numbers of essential genes compared to MAB^S^, and many of these genes encode functions important for cell wall biosynthesis and the stress response. Thus, the lack of GPL likely impacts how MAB senses environmental stresses, many of which occur during infection. It should be noted that not all the MAB^R^-specific essential genes are directly tied to environmental stress. Indeed, our data also suggest that transition to MAB^R^ rewires the bacterium’s regulatory networks and physiology independently of external stresses. For example, inactivation of MAB_3363c which encodes electron transfer flavoprotein subunit beta impacts the fitness of MAB^S^ greater than MAB^R^, while inactivation of acetylglutamate kinase (*argB*) impact fitness in MAB^R^ more than MAB^S^. A recent study showed that MAB essential genes vary between strains, with 60% of core essential genes shared (34). However, this study did not examine morphotype-specific essential genes (34).

Like many other mycobacterial species, MAB encodes two succinate:quinone oxidoreductases (Sdh1 and Sdh2), which are membrane-bound protein complexes that couple the oxidation of succinate to fumarate with the reduction of quinone to quinol in the cytoplasmic membrane. In Mtb, neither Sdh1 or Sdh2 are essential, although simultaneous CRISPRi knockdown of these enzymes revealed they are critical for optimal growth on both fermentable and nonfermentable carbon sources (86). However, in *M. smegmatis*, Sdh1 is dispensable while Sdh2 is essential due to its requirement for generating a membrane potential in hypoxic environments (83). It is not clear why Sdh2C is uniquely essential in MAB^S^, although this may be associated with the propensity of the MAB^S^ morphotype to form hypoxic biofilms (23, 72). One of the more interesting results from our study was the high frequency of suppressor mutations acquired by the MAB^R^ *sdh2C* transposon mutants, which prevented this gene from being classified as essential in MAB^R^. It is not clear why this morphotype acquires suppressor mutations at a higher frequency than MAB^S^, and it will be interesting to determine the identity and function of these mutations in the future.

We expect that the Tn-seq data generated in this study will be critical for developing new therapeutics to treat MAB infection. Phage therapy for MAB has shown promising results, but to date, its effectiveness has been demonstrated only against MAB^R^ strains (87, 88). While the presence/absence of GPL in the morphotypes was thought to be a primary reason for this differential susceptibility, Dedrick et al. showed that smooth MAB strains can be infected but are not killed (87). These data, along with the identification of non-GPL factors important for productive phage infection in mycobacteria including trehalose polyphleates biosynthetic genes (89), nucleoid-associated protein Lsr2 (90), and MoxR ATPase MSMEG_3147 (MAB_2726) (91), suggest that other physiologic factors likely contribute to phage killing. Thus, our finding of morphotype-specific essential genes may hold the clues to why MAB^S^ strains are resistant to phage killing.

Aside from phage, there are new drugs in the pipeline for treating chronic MAB infection. However, there is interest in repurposing antitubercular drugs to treat MAB infections. For example, the serine/threonine protein kinases PknA and PknB have been shown to be essential in Mtb (57), and small molecule inhibitors have been developed. Both PknA and PknB are highly conserved in MAB, and *pknB* was shown to be essential in both MAB morphotypes in vitro. These results are consistent with the results of Rifat et al., which lists *pknB* as essential, and *pknA* as having a growth defect in MAB^S^ (55). While mutation of *pknA* was not essential in MAB^S^ in vitro, mutants in this gene showed a fitness defect in both morphotypes in the abscess, thus small molecule inhibitors of PknA may be efficacious in human infections. Aside from repurposing Mtb drugs, our data indicate that while there are differences between the morphotypes, they share hundreds of essential genes that can potentially serve as therapeutic targets.

While not a cystic fibrosis lung infection model, which is a primary focus of MAB research, the abscess model is tractable, allows for two abscesses per mouse, and addresses the need for a model to study human extrapulmonary infections, which are becoming more prominent (8–14). In this model, MAB^R^ induces more inflammation and requires genes important for adaptation to stress, many of which are not important for fitness of MAB^S^ in the mouse abscess. Exposing MAB^S^ to a MAB^R^-like immune response in the abscess revealed that 16 of the 97 uniquely essential MAB^R^ genes in the abscess are likely a result of the proinflammatory response to MAB^R^. Thus, the majority of the MAB^R^ uniquely essential genes in the abscess are not mediated by the immune response to the rough morphotype, and likely are impacted by the nutritional/physical environment of the infection site. We were surprised that MAB^S^ had higher numbers of essential genes in the abscess compared to MAB^R^. These data suggest that while the host response to MAB^S^ is less proinflammatory than to MAB^R^, the smooth morphotype requires greater functionality to survive in vivo. We propose that these unique MAB^S^ in vivo essential genes may provide clues to the selective forces that govern the MAB^S^-to-MAB^R^ transition during chronic human infection.

Finally, it is important to note that we were highly conservative in our analysis of the Tn-seq data, and there are likely genes that are critical for fitness both in vitro and in vivo that we did not classify as such in this study. Longer-term infection studies will be critical to fully determine the genes required by MAB for chronic infection in this, and potentially other, murine models. Based on our studies of *Staphylococcus aureus* and *P. aeruginosa* in multiple animal models (51, 52), we anticipate that many of the essential genes identified in the abscess model will be important for fitness in other murine models, including the more commonly used lung models. Ultimately, these data provide strong support for studying gene essentiality and therapeutic development in both morphotypes to ensure maximum efficacy.

## Methods

### Strains and Growth Conditions.

*M. abscessus* ATCC19977 was obtained from American Tissue Culture Collection (ATCC). The lyophilized bacteria were resuspended per the manufactures protocol and streaked on 7H10 agar supplemented with 0.01% glycerol and 10% OADC (2.5 g Bovine Albumin Fraction V, 1 g dextrose, 0.002 g catalase, 0.025 g oleic acid, 0.425 g sodium chloride in 50 mL of water). A MAB^S^ and MAB^R^ colony was isolated from the streak plate, grown overnight, and stored at −80 °C. For our studies, MAB was grown on: 7H11 agar containing 0.5% glycerol and 10% OADC; 7H9 supplemented with 0.5% glycerol, 10% OADC, and 0.05% tween-80; or in 7H9T, which is 7H9 containing 1 g/L tryptone. Tryptone was added because this is present in 7H11 agar, which was used to generate the Tn-seq libraries. Clinical isolates were previously isolated from sputum obtained from people with CF at the Emory Cystic Fibrosis Center, and these isolated have previously been genome sequenced (7). Where indicated, antibiotics or small molecules were used at the following concentrations: Kanamycin (Km) (*Escherichia coli*: 50 μg/mL; MAB: 200 μg/mL in agar or 100 μg/mL in liquid; Zeocin 50 μg/mL), Isoniazid (IZD) 60 μg/mL, or anhydrotetracycline (ATc) 500 ng/mL. A full list of strains used in this project can be found in *SI Appendix*, Table S4.

### WGS and SNP calling.

MAB ATCC 19977 MAB^S^ and MAB^R^ were previously sequenced by our lab (7). Briefly, sequencing was done by the Microbial Sequencing and Analysis Center (MIGS, Pittsburg) or SeqCoast (New Hampshire). Short read libraries were generated using the Illumina DNA Prep kit with custom IDT 10 bp unique dual indices that target insert size of 320 bp and sequenced on an Illumina NovaSeq 6000 sequencer. Long read sequencing was generated with the Nanopore native barcoding kit and sequenced on a Nanopore platform. Genomes were assembled with Bactopia V3.0.0 (92) using—shovill_assembler spades—species “*M. abscessus*”. SNPs were identified by mapping the Illumina reads against the opposite morphotype’s genome using Breseq (93). SNPs identified by reads of the homologous genome (MAB^R^ reads to MAB^R^ assembly) were excluded from results.

### Construction of Tn-Seq Libraries.

The MycoMar phage was provided by the Rubin lab (94). For transposon library construction, MAB strains were subcultured in 50 mL of 7H9T media and grown to mid-log phase. Bacteria were pelleted and washed twice with prewarmed (37 °C) MP buffer (50 mM Tris–HCl, pH 7.5,150 mM NaCl, 10 mM MgSO_4_, 2 mM CaCl_2_), and resuspended in 5 mL of warm MP. A 100 μL aliquot was removed and spread onto 7H11+Km agar plates as a control. The remaining bacteria were inoculated with 0.5 to 1 × 10^11^ plaque forming units of the MycoMar phage and incubated at 37 °C for 4 h. Phage-treated bacteria were then serial diluted and spread on 7H11+Km plates to select for transposon mutants. The remaining phage-treated bacteria were stored in 10% glycerol at −80 °C. Once the transduction efficiency was determined from the serially diluted cells, frozen transposon libraries were thawed and an appropriate amount spread onto 245 mm square 7H11+Km agar plates (Sigma, St. Louis, MO) to ensure separation of transposon mutant colonies. Plates were incubated at 37 °C for 7 d, after which colonies were combined from multiple plates to create the transposon mutant pools. Pooled mutants were gently homogenized with 7H9T media and glycerol to disrupt any clumps then aliquoted and stored as aliquots at −80 °C.

### Sequencing of Tn-Seq Libraries.

To create the transposon sequencing libraries, DNA was extracted from individual tubes of frozen transposon libraries, 5 mL in vitro grown cultures, or abscess infections. For each type of sample bacteria was pelleted at 4,000 g for 5 min and suspended in 250 μL 10 mM Tris, 1 mM EDTA, pH 8.0 in BeadBug tubes with 0.1 mm acid-washed silica glass beads (Sigma-Aldrich, St. Louis, MO). The samples were mechanically lysed using 3 rounds of bead-beating with the Mini-Beadbeater-16 (Biospec Products, Bartlesville, OK) for 30 s, with a 2-min incubation on ice between each round. Samples were then enzymatically lysed with 30 μL of 10 mg/mL lysozyme and incubated for 2 h at 37 °C. 4 μL of RNase A (10 mg/mL) was then added, and samples were incubated for an additional hour at 37 °C. 20 μL Proteinase K (20 mg/mL) and sodium dodecyl sulfate (SDS) to a final concentration of 3.33% were added, and samples were incubated at 37 °C for 2 h. Finally, Proteinase K was heat killed for 10 min at 95 °C. An equal volume of phenol:chloroform:isoamyl alcohol (25:24:1, pH 8.0) was then added to each sample, vortexed for 1 min, and then centrifuged at 12,000×*g* for 5 min. The aqueous phase was precipitated by addition of 0.1 volume of 3 M sodium acetate and 0.8 volume 100% isopropanol, mixed, and stored at −20 °C for at least 30 min. DNA was pelleted by centrifuging at 4 °C for 30 min, washed twice with 1 mL 75% ethanol, and dissolved in 100 µL water.

Isolated DNA was prepared for transposon sequencing following a previously published protocol (see Tn-Seq Illumina Library Preparation from Initial PA14 Mutant Library in supplemental material for ref. 48) with the following modifications: 1) MycoMar specific primers were used (*SI Appendix*, Table S5), 2) between steps, DNA was purified using the GeneJet PCR purification kit (Thermo Scientific, Waltham, MA); 3) 2 to 4 PCR-1 reactions were performed, each with 0.5 to 1 µg DNA and pooled in the following purification step; 4) PCR-2 was carried out with a mixture of 4 primers containing 3, 5, 7, or 9 random bases to increase the sequence diversity of our libraries; and 5) DNA size selection after PCR-2 was performed as described in the NEB “QC Check and Size Selection using 6% PolyAcrylamide Gel - NEBNext Multiplex Small RNA Sample Prep Set for Illumina” protocol. DNA was cut out between the 160 bp and 307 bp ladder markers and isolated (95). Libraries were pooled and sequenced at the Georgia Tech Molecular Evolution Core using a NextSeq 500 sequencer (Illumina, San Diego, CA) with 1 × 75 v2 chemistry.

### Tn-Seq Read Processing, Mapping, and Essentials Analysis.

All analysis scripts are adapted from previously published protocols (50, 51, 96) and are available at https://github.com/brross60/Tn-seq.git. TnSeq3-2.sh was used. This script selects for reads possessing the MycoMar Inverted Repeat (IR) (CAACCTGT) using Cutadapt v3.3, maps the reads to MAB ATCC 19977 (GCF_000069185.1) with Bowtie2 v2.4.2, and assigns each read to an insertion site. Occupied sites were defined as those with ≥.3 reads/million reads as >90% of sites are TA sites. Essential genes were determined using TnSeqDESeq2Essential_mariner.sh and TnGeneBin.pl relying on R v3.4.2, DESeq2 v1.18.1 (59), and mclust v5.4. This script was run with the following parameters: 1,000 expected pseudodatasets, trim = 0 (none of the most abundant sites were removed from the analysis), no LOESS smoothing (as it did not impact the results), and no site filtering (all transposon insertion sites were analyzed, no matter the number of replicates they were identified in). This essentials analysis normalized samples for sequencing depth using DESeq2 estimateSizeFactors() (97). Pseudodatasets were constructed with the same number of insertion sites and total reads mapping as the average of the experimental replicates, randomly distributed across the genome at TA sites. Next, insertion sites in the experimental and pseudodatasets were binned by gene using a modified gff lacking 10% of the 3’ end of each CDS (to ignore insertions at the 3’ end of a gene that may not impact function). DESeq2 was used to compare the mutant abundances in the experimental data to the mutant abundances in pseudodatasets with estimateDispersons() run with fitType=“local.” We ran nbinomWaldTest() with betaPrior = TRUE. betaPrior = TRUE was used as this shrinks log_2_ fold-changes when counts are low, dispersion is high, or degrees-of-freedom are low, and this is conservative. Genes were called “essential” if the log_2_ fold-change and adjusted *P*-value (Benjamini–Hochberg adjusted *P*-value, negative binomial Wald test in DESeq2) were less than or equal to the maximum values obtained for 22 genes previously defined as universally essential in bacteria (54). MAB^S^ and MAB^R^ essential genes in the library stocks were further restricted to genes that had ≥4-fold difference in read abundance and a *P*-value of ≤0.05 as defined by TnSeqDESeq2_DifferentialsAnalysis.R Genes that were essential by Monte Carlo but did not meet the differential frequency requirements were deemed “ambiguous”. For analysis of essential genes at mid-log phase, essential genes were defined by Monte Carlo. Genes essential in the frozen stocks of the respective morphotype were removed. Genes with a fitness defect are those with a log_2_ fold-change of ≤−2 and *P*-value of 0.001. Differences between essential and nonessential gene sizes were determined using the Kruskal–Wallis test.

Abscess Tn-seq samples were clustered by first normalizing by size factor, variance stabilizing transforming the data (DeSeq2 v1.44.0), then plotting as a Principal Component plot via pcaPlot(). Genes essential in the abscess were determined by Monte Carlo. Genes essential in the frozen stocks, at mid-log, or with genes with ≤10 normalized reads in the inoculum of the respective morphotype were removed. Before comparing MAB^S^ and MAB^R^ abscess essential genes, in vitro essential genes were removed. The Venn diagrams were generated using Venny (v2.1) (40).

### CRISPRi Repression of Essential Genes.

pILR2 was obtained from Addgene and guide RNAs cloned into the plasmid following a previously published protocol (98). Briefly, sgRNAs were selected from sgRNA Design Tool for Mycobacteria v2.0, selecting for primer pairs with the highest predicted strength and closest to the 5’ end of the gene (*SI Appendix*, Table S5) (98). Primers were ordered from Integrated DNA Technologies (Coralville, IA). sgRNA-encoding fragments were generated by reconstituting primers to 100 μM, mixed at equal parts in annealing buffer (50 mM Tris pH 7.5, 50 mM NaCl, 1 mM EDTA) and incubating in a thermocycler at 95 °C for 2 min and reducing heat −0.1 °C /s to 25 °C. pILR2 was restriction digested with BsmBI (NEB, Ipswich, MA). The DNA encoding the sgRNA and plasmid were PCR purified with the Qiagen Miniprep kit (Qiagen, Hilden, Germany), Gibson assembled (NEB, Ipswich, MA), and subcloned in *E. coli* DH5α (NEB, Ipswich, MA). Plasmids were confirmed by PCR and sequencing. CRISPRi plasmids were electroporated into MAB^S^ and MAB^R^ electrocompetent cells and transformants selected for on kanamycin 50 μg/mL 7H11 agar. Isolates were confirmed by PCR and stored at −80 °C. To test the effect of CRISPRi induction on survival, disc diffusion, dot plates, or liquid growth was done. *Disc Diffusion*: 7H11 plates were spread with MAB containing a CRISPRi plasmid (100 μL of 0.05 OD_600_), and a disc containing 10,000 ng of ATc or an equal volume of vehicle control was placed onto the agar surface. Plates were incubated at 37 °C and discs supplemented with ATc daily to account for its 24-hour half-life and imaged after 96 h. *Dot plates*: Stationary phase MAB were normalized to an OD_600_ of 0.25 (10^8^ CFU/mL) was 10-fold diluted in a 96-well plate and 10ul of each dilution plated on plates with or without 500 ng/mL of ATc. *Liquid growth*: Bacteria were grown in 7H9T then subcultured to an OD_600_ of 0.05 with 1000 ng/mL ATc or an equal volume of vehicle control (DMSO). Cultures were grown shaking at 150 rpm in 37 °C, supplementing ATc daily to account for its 24-h half-life, and cultures were imaged with an Android phone after 48 h. Alternatively, 7H11 plates were spread with MAB containing a CRISPRi plasmid (100 μL of 0.05 OD_600_), and a disc containing 1000 ng of ATc or an equal volume of vehicle control was placed onto the agar surface. Plates were incubated at 37 °C and discs supplemented with ATc daily to account for its 24-hour half-life and imaged after 96 h.

### qRT-PCR.

Stationary phase cultures of MAB strains carrying pILR2 expressing an sgRNA or vector control were subcultured and grown to mid-log. Cultures were then diluted to an OD_600_ of 0.05 in the presence of 500 ng/μL of ATc and grown shaking for 16 h. Bacterial cells were pelleted by centrifugation and pellets resuspended in TRIzol (Invitrogen, Waltham, MA). RNA was isolated using the manufacturer’s protocol with the exception that samples were mechanically lysed using 3 rounds of bead-beating with the Mini-Beadbeater-16 (Biospec Products, Bartlesville, OK) for 30 s, with a 2-min incubation on ice between each round. RNA was DNAase treated (Promega, Madison, WI) and further purified using TRIzol reagent and precipitation. Luna One-Step RT-qPCR (NEB, Ipswich, MA) was used with 100 ng of RNA and gene specific primers for *pknA*, *pknB*, and *sigA* (*SI Appendix*, Table S5). Gene expression was normalized using *sigA* as previously described (57) and significance calculated using the Mann–Whitney test.

### Mutant Generation.

The MAB *pknA* mutant was generated as previously described (99). Briefly, a fragment was generated containing a concatenation of 1000 bp of the upstream and 1886 bp of the downstream regions of *pknA* (*SI Appendix*, Table S5). The purified PCR fragments were assembled into pUX1-katG using Gibson Assembly (NEB, Ipswich, MA) and subcloned in *E. coli* Dh5α to yield pUX::ΔpknA. The plasmid was isolated and confirmed by whole plasmid sequencing by Plasmidsaurus (San Francisco, CA). Logarithmic MAB cultures were chilled, washed three times in 10% glycerol, and resuspended in 10% of the original volume. 200 μL of chilled cells were electroporated with 1 μg of pUX::Δ*pknA*. Electroporated bacteria were grown in 7H9 overnight and plated on 7H10 plates containing kanamycin. pUX1-katG contains tdTomato and merodiploid fluorescent red colonies were chosen, grown in kanamycin media, subcultured without antibiotics for 4 h, and spread on 7H10 plates containing isoniazid to select for plasmid excision. Mutants were validated by PCR and sequencing by Plasmidsaurus (San Francisco, CA). MAB strains containing deletion of MAB_3690 or MAB_2606c were generated by allelic exchange using amplicons containing 950 bp upstream DNA, a zeocin resistance gene, and 950 bp DNA downstream of the respective genes. These amplicons were electroporated into MAB^S^ containing the recombinase expressing plasmid pJV53 and selected using kanamycin and zeocin. Resulting mutants were cultured until pJV53 was lost (kanamycin sensitive) and the mutation confirmed via genome sequencing (Plasmidsaurus).

### Abscess Infection.

This study was carried out in strict accordance with the recommendations in the Guide for the Care and Use of Laboratory Animals of the National Institutes of Health. Animal protocols were approved by the Institutional Animal Care and Use Committees of Georgia Institute of Technology (protocol no. A100127). For WT infections, bacteria were grown on 7H11 plates followed by culturing in supplemented 7H9T media shaking at 150 RPM at 37 °C for 3 d. Bacteria were washed and adjusted to 1 × 10^7^ CFU/dose endotoxin free PBS prior to infection. For Tn-seq infections, stocks of the MAB^S^ or MAB^R^ transposon mutant libraries were grown aerobically overnight at 37 °C to mid-log (OD_600_ 0.8 to 1.0). Cells were washed and normalized to 5 × 10^7^ CFU/dose in PBS. For abscess infections that contained heat killed MAB^R^ bacteria, washed MAB^R^ cells were resuspended in PBS to and heat killed at 95 °C for 15 min. Killed bacteria were cooled to room temperature and mixed in equal parts with the live MAB^S^ transposon library to yield 5 × 10^7^ CFU/dose of live bacteria and heat killed bacteria. A higher live bacterial number was used in these studies to avoid potential bottlenecking of the Tn library by immune activation with heat killed MAB^R^.

For coinfection studies with MAB deletion mutants, WT MAB^S^ (zeocin sensitive) was mixed with ΔMAB_3690 (zeocin resistant) or ΔMAB_2606c (zeocin resistant) in the presence of HK MAB^R^. 5 × 10^7^ CFU of live bacteria (2.5 × 10^7^ CFU of WT and one of the mutants) were used to initiate the infection. Abscesses were collected at 3 d postinfection, serial diluted, and plated on 7H10 with and without zeocin to quantify mutant and WT CFUs respectively. WT burden was determined by subtracting zeocin resistant CFUs from total CFUs (no zeomycin).

Nair was used to remove hair from both inner thighs of 6 to 8-week-old Swiss Webster mice (Charles River, Wilmington, MA). 70% ethanol was used to sterilize the area of injection. Abscesses were initiated by subcutaneous injection of 100 µL into each inner thigh. Animals were euthanized and abscesses were harvested after 3 d. Abscesses were homogenized in PBS by beating for 30 s in BeadBug tubes (Sigma-Aldrich, St. Louis, MO) with 2.8-mm steel beads using a Mini-Beadbeater-16 (BioSpec Products, Bartlesville, OK). Microbial abundance was determined by calculating colony-forming units (CFUs) after dilution plating on 7H11 agar and incubating for 3 to 5 d at 37 °C. Differences in CFUs and abscess weight were assessed by the Mann–Whitney test on GraphPad v10.3.0. The remaining homogenate was processed for cytokine and chemokine analysis (see respective methods section). For Tn-seq, bacterial pellets were resuspended in 4.5 mL 7H9T+ 40 μg/mL kanamycin and grown at 37 °C for 8 h then frozen at −80 °C until DNA isolates (please see the “*Sequencing of Tn-Seq Libraries”* methods section).

### Cytokine and Chemokine Analysis.

Abscess supernatants were centrifuged and further clarified by spinning at 16,000 g at 4 °C for 30 min. Then, 200 μL of supernatant was flash frozen and stored at −80 °C until analysis. Samples were processed by the Emory Multiplex Immunoassay Core using the V-PLEX Proinflammatory Panel1 (mouse) Kit (Meso Scale Diagnostics, Rockville, MD) following the manufactures protocol. The raw data were provided as pg/mL. To obtain pg/mg the raw data was multiplied by 0.45 to get pg/abscess then divided by the abscess weight to obtain pg/mg. This data were analyzed using one-way ANOVA with Kruskal–Wallis correction on GraphPad v10.3.0.

## Supplementary Material

Appendix 01 (PDF)

Dataset S01 (XLSX)

Dataset S02 (XLSX)

Dataset S03 (XLSX)

Dataset S04 (XLSX)

Dataset S05 (XLSX)

Dataset S06 (XLSX)

## Data Availability

Sequencing data have been deposited in SRA (PRJNA1190882). A list of all samples used in this work can be found in *SI Appendix*, Table S2. All other data are included in the manuscript and/or supporting information. Previously published data were used for this work (7, 55).

## References

[1] V. Deretic, M. J. Schurr, H. Yu, *Pseudomonas aeruginosa*, mucoidy and the chronic infection phenotype in cystic fibrosis. Trends Microbiol. **3**, 351–356 (1995). 8520888 10.1016/s0966-842x(00)88974-x

[2] L. Goltermann, T. Tolker-Nielsen, Importance of the exopolysaccharide matrix in antimicrobial tolerance of *Pseudomonas aeruginosa* aggregates. Antimicrob. Agents Chemother. **61**, e02696-16 (2017). 28137803 10.1128/AAC.02696-16 PMC5365683

[3] L. M. Simpson, V. K. White, S. F. Zane, J. D. Oliver, Correlation between virulence and colony morphology in *Vibrio vulnificus*. Infect. Immun. **55**, 269–272 (1987). 2432016 10.1128/iai.55.1.269-272.1987 PMC260315

[4] T. Nishimura , The rough colony morphotype of *Mycobacterium avium* exhibits high virulence in human macrophages and mice. J. Med. Microbiol. **69**, 1020–1033 (2020). 32589124 10.1099/jmm.0.001224

[5] S. Besier , Prevalence and clinical significance of *Staphylococcus aureus* small-colony variants in cystic fibrosis lung disease. J. Clin. Microbiol. **45**, 168–172 (2007). 17108072 10.1128/JCM.01510-06 PMC1828983

[6] D. J. Wolter , *Staphylococcus aureus* small-colony variants are independently associated with worse lung disease in children with cystic fibrosis. Clin. Infect. Dis. **57**, 384–391 (2013). 23625938 10.1093/cid/cit270 PMC3888146

[7] B. N. Ross, E. Evans, M. Whiteley, Phenylacetic acid metabolic genes are associated with *Mycobacteroides abscessus* dominant circulating clone 1. Microbiol. Spectr. **12**, e0133024 (2024), 10.1128/spectrum.01330-24. 39315786 PMC11537035

[8] D. S. Tokunaga, A. M. Siu, S. Y. Lim, Nontuberculous mycobacterial skin and soft tissue infection in Hawai’i. BMC Infect. Dis. **22**, 360 (2022). 35410188 10.1186/s12879-022-07345-y PMC9004129

[9] K. Kavitha , The burden of mycobacteria species among children from postvaccination abscess in southern India. Int. J. Mycobacteriol. **10**, 358–363 (2021). 34916452 10.4103/ijmy.ijmy_190_21

[10] C. F. Chen , *Mycobacterium abscessus* infection after facial injection of argireline: A case report. World J. Clin. Cases **9**, 1996–2000 (2021). 33748252 10.12998/wjcc.v9.i8.1996 PMC7953399

[11] M. A. Davis, S. Antony, T9–T10 osteomyelitis, epidural abscess and cord compression secondary to *Mycobacterium abscessus*: A case report. Infect. Disord. **21**, 289–293 (2021). 10.2174/1871526520666200428095022 32342821

[12] K. Bala, S. Kumari, R. Guleria, U. Singh, Recurrent bilateral breast abscess due to *Mycobacterium abscessus* in an immune-competent woman. BMJ Case Rep. **13**, e235857 (2020). 10.1136/bcr-2020-235857 PMC7604781 33127731

[13] A. A. Leto Barone , Atypical mycobacterial infections after plastic surgery procedures abroad: A multidisciplinary algorithm for diagnosis and treatment. Ann. Plast. Surg. **84**, 257–262 (2020). 31688120 10.1097/SAP.0000000000002061

[14] R. M. Dedrick , Engineered bacteriophages for treatment of a patient with a disseminated drug-resistant Mycobacterium abscessus. Nat. Med. **25**, 730–733 (2019). 31068712 10.1038/s41591-019-0437-z PMC6557439

[15] R. Chadha , An outbreak of post-surgical wound infections due to *Mycobacterium abscessus*. Pediatr. Surg. Int. **13**, 406–410 (1998). 9639628 10.1007/s003830050350

[16] A. W. Baker , Two-phase hospital-associated outbreak of *Mycobacterium abscessus*: Investigation and mitigation. Clin. Infect. Dis. **64**, 902–911 (2017). 28077517 10.1093/cid/ciw877 PMC5848312

[17] R. Thomson, C. Tolson, H. Sidjabat, F. Huygens, M. Hargreaves, *Mycobacterium abscessus* isolated from municipal water—a potential source of human infection. BMC Infect. Dis. **13**, 241 (2013). 23705674 10.1186/1471-2334-13-241 PMC3668184

[18] J. N. Sinkevitch , Notes from the field: *Mycobacterium abscessus* outbreak related to contaminated water among ventilator-dependent residents of a pediatric facility - Pennsylvania, 2022. MMWR. Morb. Mortal. Wkly. Rep. **72**, 1151–1152 (2023). 37856361 10.15585/mmwr.mm7242a5 PMC10602627

[19] K. M. Kreutzfeldt , Molecular longitudinal tracking of *Mycobacterium abscessus* spp. during chronic infection of the human lung. PLoS One **8**, e63237 (2013). 23696800 10.1371/journal.pone.0063237 PMC3655965

[20] E. Catherinot , Acute respiratory failure involving an R variant of *Mycobacterium abscessus*. J. Clin. Microbiol. **47**, 271–274 (2009). 19020061 10.1128/JCM.01478-08 PMC2620830

[21] J. Y. Kam , Rough and smooth variants of Mycobacterium abscessus are differentially controlled by host immunity during chronic infection of adult zebrafish. Nat. Commun. **13**, 952 (2022). 35177649 10.1038/s41467-022-28638-5 PMC8854618

[22] E. Catherinot , Hypervirulence of a rough variant of the *Mycobacterium abscessus* type strain. Infect. Immun. **75**, 1055–1058 (2007). 17145951 10.1128/IAI.00835-06 PMC1828507

[23] S. T. Howard , Spontaneous reversion of Mycobacterium abscessus from a smooth to a rough morphotype is associated with reduced expression of glycopeptidolipid and reacquisition of an invasive phenotype. Microbiology (Reading) **152**, 1581–1590 (2006). 16735722 10.1099/mic.0.28625-0

[24] L. J. Caverly , *Mycobacterium abscessus* morphotype comparison in a murine model. PLoS One **10**, e0117657 (2015). 25675351 10.1371/journal.pone.0117657 PMC4326282

[25] K. Ryan, T. F. Byrd, *Mycobacterium abscessus*: Shapeshifter of the mycobacterial world. Front. Microbiol. **9**, 2642 (2018). 30443245 10.3389/fmicb.2018.02642 PMC6221961

[26] P. Supply , Genomic analysis of smooth tubercle bacilli provides insights into ancestry and pathoadaptation of Mycobacterium tuberculosis. Nat. Genet. **45**, 172–179 (2013). 23291586 10.1038/ng.2517 PMC3856870

[27] M. Orgeur, C. Sous, J. Madacki, R. Brosch, Evolution and emergence of *Mycobacterium tuberculosis*. FEMS Microbiol. Rev. **48**, fuae006 (2024). 38365982 10.1093/femsre/fuae006 PMC10906988

[28] J. M. Bryant , Emergence and spread of a human-transmissible multidrug-resistant nontuberculous mycobacterium. Science **354**, 751–757 (2016). 27846606 10.1126/science.aaf8156 PMC5142603

[29] J. M. Bryant , Stepwise pathogenic evolution of *Mycobacterium abscessus*. Science **372**, eabb8699 (2021). 33926925 10.1126/science.abb8699 PMC7611193

[30] N. Commins , Mutation rates and adaptive variation among the clinically dominant clusters of Mycobacterium abscessus. Proc. Natl. Acad. Sci. U.S.A. **120**, e2302033120 (2023). 37216535 10.1073/pnas.2302033120 PMC10235944

[31] M. Hogardt, J. Heesemann, Adaptation of *Pseudomonas aeruginosa* during persistence in the cystic fibrosis lung. Int. J. Med. Microbiol. **300**, 557–562 (2010). 20943439 10.1016/j.ijmm.2010.08.008

[32] A. J. Carterson , The transcriptional regulator AlgR controls cyanide production in *Pseudomonas aeruginosa*. J. Bacteriol. **186**, 6837–6844 (2004). 15466037 10.1128/JB.186.20.6837-6844.2004 PMC522194

[33] B. Lambert , A novel phase variant of the cholera pathogen shows stress-adaptive cryptic transcriptomic signatures. BMC Genomics **17**, 914 (2016). 27842489 10.1186/s12864-016-3233-x PMC5109742

[34] C. Akusobi , Transposon-sequencing across multiple *Mycobacterium abscessus* isolates reveals significant functional genomic diversity among strains. mBio **16**, e0337624 (2025). 39745363 10.1128/mbio.03376-24 PMC11796383

[35] L. Laencina , Identification of genes required for *Mycobacterium abscessus* growth in vivo with a prominent role of the ESX-4 locus. Proc. Natl. Acad. Sci. U.S.A. **115**, E1002–E1011 (2018). 29343644 10.1073/pnas.1713195115 PMC5798338

[36] M. R. Sullivan , Biotin-dependent cell envelope remodelling is required for *Mycobacterium abscessus* survival in lung infection. Nat. Microbiol. **8**, 481–497 (2023). 36658396 10.1038/s41564-022-01307-5 PMC9992005

[37] R. Rodriguez , MarR-Dependent transcriptional regulation of mmpSL5 induces ethionamide resistance in mycobacterium abscessus. Antimicrob. Agents. Chemother. **67**, e0135022 (2023). 36988462 10.1128/aac.01350-22 PMC10112066

[38] S. Wang , Arabinosyltransferase c mediates multiple drugs intrinsic resistance by altering cell envelope permeability in *Mycobacterium abscessus*. Microbiol. Spectr. **10**, e0276321 (2022). 35946941 10.1128/spectrum.02763-21 PMC9430846

[39] C. Akusobi , Transposon mutagenesis in *Mycobacterium abscessus* identifies an essential penicillin-binding protein involved in septal peptidoglycan synthesis and antibiotic sensitivity. eLife **11**, e71947 (2022). 35659317 10.7554/eLife.71947 PMC9170245

[40] B. Shanmugham, A. Pan, Identification and characterization of potential therapeutic candidates in emerging human pathogen *Mycobacterium abscessus*: A novel hierarchical in silico approach. PLoS One **8**, e59126 (2013). 23527108 10.1371/journal.pone.0059126 PMC3602546

[41] X. Carette , Multisystem analysis of *Mycobacterium tuberculosis* reveals kinase-dependent remodeling of the pathogen-environment interface. mBio **9**, e02333-17 (2018). 29511081 10.1128/mBio.02333-17 PMC5845002

[42] T. Wang , Mtb PKNA/PKNB dual inhibition provides selectivity advantages for inhibitor design to minimize host kinase interactions. ACS Med. Chem. Lett. **8**, 1224–1229 (2017). 29259738 10.1021/acsmedchemlett.7b00239 PMC5733270

[43] M. Moore, J. B. Frerichs, An unusual acid-fast infection of the knee with subcutaneous, abscess-like lesions of the gluteal region; report of a case with a study of the organism, Mycobacterium abscessus, n. sp. J. Invest. Dermatol. **20**, 133–169 (1953). 13035193 10.1038/jid.1953.18

[44] C. Ruis , Dissemination of *Mycobacterium abscessus* via global transmission networks. Nat. Microbiol. **6**, 1279–1288 (2021). 34545208 10.1038/s41564-021-00963-3 PMC8478660

[45] A. Pawlik , Identification and characterization of the genetic changes responsible for the characteristic smooth-to-rough morphotype alterations of clinically persistent *Mycobacterium abscessus*. Mol. Microbiol. **90**, 612–629 (2013). 23998761 10.1111/mmi.12387

[46] A. V. Gutierrez, A. Viljoen, E. Ghigo, J. L. Herrmann, L. Kremer, Glycopeptidolipids, a double-edged sword of the *Mycobacterium abscessus* complex. Front. Microbiol. **9**, 1145 (2018). 29922253 10.3389/fmicb.2018.01145 PMC5996870

[47] M. S. Siegrist, E. J. Rubin, Phage transposon mutagenesis. Methods Mol. Biol. **465**, 311–323 (2009). 20560067 10.1007/978-1-59745-207-6_21

[48] K. H. Turner, A. K. Wessel, G. C. Palmer, J. L. Murray, M. Whiteley, Essential genome of Pseudomonas aeruginosa in cystic fibrosis sputum. Proc. Natl. Acad. Sci. U.S.A. **112**, 4110–4115 (2015). 25775563 10.1073/pnas.1419677112 PMC4386324

[49] A. M. Narayanan, M. M. Ramsey, A. Stacy, M. Whiteley, Defining genetic fitness determinants and creating genomic resources for an oral pathogen. Appl. Environ. Microbiol. **83**, e00797-17 (2017). 28476775 10.1128/AEM.00797-17 PMC5494627

[50] G. R. Lewin, A. Stacy, K. L. Michie, R. J. Lamont, M. Whiteley, Large-scale identification of pathogen essential genes during coinfection with sympatric and allopatric microbes. Proc. Natl. Acad. Sci. U.S.A. **116**, 19685–19694 (2019). 31427504 10.1073/pnas.1907619116 PMC6765283

[51] C. B. Ibberson , Co-infecting microorganisms dramatically alter pathogen gene essentiality during polymicrobial infection. Nat. Microbiol. **2**, 17079 (2017). 28555625 10.1038/nmicrobiol.2017.79 PMC5774221

[52] K. H. Turner, J. Everett, U. Trivedi, K. P. Rumbaugh, M. Whiteley, Requirements for *Pseudomonas aeruginosa* acute burn and chronic surgical wound infection. PLoS Genet. **10**, e1004518 (2014). 25057820 10.1371/journal.pgen.1004518 PMC4109851

[53] J. L. Murray, T. Kwon, E. M. Marcotte, M. Whiteley, Intrinsic antimicrobial resistance determinants in the superbug *Pseudomonas aeruginosa*. MBio **6**, e01603–01615 (2015). 26507235 10.1128/mBio.01603-15 PMC4626858

[54] D. Shaw, A. Hermoso, M. Lluch-Senar, L. Serrano, Comparative gene essentiality across the bacterial domain. bioRxiv [Preprint] (2020). 10.1101/2020.02.28.969238 (Accessed 5 March 2024).

[55] D. Rifat, L. Chen, B. N. Kreiswirth, E. L. Nuermberger, Genome-wide essentiality analysis of *Mycobacterium abscessus* by saturated transposon mutagenesis and deep sequencing. MBio **12**, e0104921 (2021). 34126767 10.1128/mBio.01049-21 PMC8262987

[56] J. M. Rock , Programmable transcriptional repression in mycobacteria using an orthogonal CRISPR interference platform. Nat. Microbiol. **2**, 16274 (2017). 28165460 10.1038/nmicrobiol.2016.274 PMC5302332

[57] J. Zeng , Protein kinases PknA and PknB independently and coordinately regulate essential Mycobacterium tuberculosis physiologies and antimicrobial susceptibility. PLoS Pathog. **16**, e1008452 (2020). 32255801 10.1371/journal.ppat.1008452 PMC7164672

[58] J. Singh , Invasive *Mycobacterium abscessus* outbreak at a pediatric dental clinic. Open Forum Infect. Dis. **8**, ofab165 (2021). 34113683 10.1093/ofid/ofab165 PMC8186244

[59] S. J. Koh , An outbreak of skin and soft tissue infection caused by Mycobacterium abscessus following acupuncture. Clin. Microbiol. Infect. **16**, 895–901 (2010). 19694761 10.1111/j.1469-0691.2009.03026.x

[60] M. I. Newman, A. E. Camberos, J. Ascherman, *Mycobacteria abscessus* outbreak in US patients linked to offshore surgicenter. Ann. Plast. Surg. **55**, 107–110; discussion 110 (2005). 15985802 10.1097/01.sap.0000168030.87804.93

[61] C. Viana-Niero , Molecular characterization of Mycobacterium massiliense and Mycobacterium bolletii in isolates collected from outbreaks of infections after laparoscopic surgeries and cosmetic procedures. J. Clin. Microbiol. **46**, 850–855 (2008). 18174307 10.1128/JCM.02052-07 PMC2268380

[62] M. Klompas , *Mycobacterium abscessus* cluster in cardiac surgery patients potentially attributable to a commercial water purification system. Ann. Intern. Med. **176**, 333–339 (2023). 36877966 10.7326/M22-3306

[63] P. Chirasuthat, K. Triyangkulsri, S. Rutnin, K. Chanprapaph, V. Vachiramon, Cutaneous nontuberculous mycobacterial infection in Thailand: A 7-year retrospective review. Medicine (Baltimore) **99**, e19355 (2020). 32150075 10.1097/MD.0000000000019355 PMC7478711

[64] F. Farid , Vertebral osteomyelitis due to *Mycobacterium abscessus* subsp. massiliense with paravertebral abscess: A case report and review. J. Infect. Chemother. **29**, 922–926 (2023). 37244350 10.1016/j.jiac.2023.05.020

[65] A. J. Beech , *Mycobacterium abscessus* skin and soft tissue infection following autologous fat grafting in Kurdistan treated with an antibiotic combination including Imipenem-Relebactam and Rifabutin. J. Clin. Tuberc. Other Mycobact. Dis. **32**, 100381 (2023). 37323244 10.1016/j.jctube.2023.100381 PMC10267594

[66] A. L. Roux , Overexpression of proinflammatory TLR-2-signalling lipoproteins in hypervirulent mycobacterial variants. Cell Microbiol. **13**, 692–704 (2011). 21143571 10.1111/j.1462-5822.2010.01565.x

[67] E. R. Rhoades , *Mycobacterium abscessus* glycopeptidolipids mask underlying cell wall phosphatidyl-myo-inositol mannosides blocking induction of human macrophage TNF-alpha by preventing interaction with TLR2. J. Immunol. **183**, 1997–2007 (2009). 19596998 10.4049/jimmunol.0802181

[68] N. Ruangkiattikul , Type I interferon induced by TLR2-TLR4-MyD88-TRIF-IRF3 controls *Mycobacterium abscessus* subsp. *abscessus* persistence in murine macrophages via nitric oxide. Int. J. Med. Microbiol. **309**, 307–318 (2019). 31178418 10.1016/j.ijmm.2019.05.007

[69] T. S. Kim, Y. S. Kim, H. Yoo, Y. K. Park, E. K. Jo, *Mycobacterium massiliense* induces inflammatory responses in macrophages through Toll-like receptor 2 and c-Jun N-terminal kinase. J. Clin. Immunol. **34**, 212–223 (2014). 24402617 10.1007/s10875-013-9978-y PMC3937545

[70] B. R. Kim, B. J. Kim, Y. H. Kook, *Mycobacterium abscessus* infection leads to enhanced production of type 1 interferon and NLRP3 inflammasome activation in murine macrophages via mitochondrial oxidative stress. PLoS Pathog. **16**, e1008294 (2020). 32210476 10.1371/journal.ppat.1008294 PMC7094820

[71] A. Bernut , *Mycobacterium abscessus*-induced granuloma formation is strictly dependent on TNF signaling and neutrophil trafficking. PLoS Pathog. **12**, e1005986 (2016). 27806130 10.1371/journal.ppat.1005986 PMC5091842

[72] R. Nessar, J. M. Reyrat, L. B. Davidson, T. F. Byrd, Deletion of the mmpL4b gene in the Mycobacterium abscessus glycopeptidolipid biosynthetic pathway results in loss of surface colonization capability, but enhanced ability to replicate in human macrophages and stimulate their innate immune response. Microbiology (Reading) **157**, 1187–1195 (2011). 21292749 10.1099/mic.0.046557-0

[73] S. Nambi , The oxidative stress network of *Mycobacterium tuberculosis* reveals coordination between radical detoxification systems. Cell Host Microbe **17**, 829–837 (2015). 26067605 10.1016/j.chom.2015.05.008 PMC4465913

[74] B. D. Kana , The resuscitation-promoting factors of *Mycobacterium tuberculosis* are required for virulence and resuscitation from dormancy but are collectively dispensable for growth in vitro. Mol. Microbiol. **67**, 672–684 (2008). 18186793 10.1111/j.1365-2958.2007.06078.x PMC2229633

[75] D. R. Sherman , Regulation of the *Mycobacterium tuberculosis* hypoxic response gene encoding alpha -crystallin. Proc. Natl. Acad. Sci. U.S.A. **98**, 7534–7539 (2001). 11416222 10.1073/pnas.121172498 PMC34703

[76] D. M. Roberts, R. P. Liao, G. Wisedchaisri, W. G. Hol, D. R. Sherman, Two sensor kinases contribute to the hypoxic response of *Mycobacterium tuberculosis*. J. Biol. Chem. **279**, 23082–23087 (2004). 15033981 10.1074/jbc.M401230200 PMC1458500

[77] N. Naor, E. Zarbib, D. Barkan, *Mycobacterium abscessus* tetracycline-modifying monooxygenase. Microbiol. Spectr. **10**, e0234622 (2022). 35894619 10.1128/spectrum.02346-22 PMC9430549

[78] H. Chandra, M. K. Gupta, Y. W. Lam, J. S. Yadav, Predominantly orphan secretome in the lung pathogen *Mycobacterium abscessus* revealed by a multipronged growth-phase-driven strategy. Microorganisms **12**, 378 (2024). 38399782 10.3390/microorganisms12020378 PMC10892769

[79] D. Mielecki , *Pseudomonas putida* AlkA and AlkB proteins comprise different defense systems for the repair of alkylation damage to DNA - In vivo, in vitro, and in silico studies. PLoS One **8**, e76198 (2013). 24098441 10.1371/journal.pone.0076198 PMC3788762

[80] P. Gorla , MtrA response regulator controls cell division and cell wall metabolism and affects susceptibility of mycobacteria to the first line antituberculosis drugs. Front. Microbiol. **9**, 2839 (2018). 30532747 10.3389/fmicb.2018.02839 PMC6265350

[81] B. S. Simcox, B. R. Tomlinson, L. N. Shaw, K. H. Rohde, Mycobacterium abscessus DosRS two-component system controls a species-specific regulon required for adaptation to hypoxia. Front. Cell. Infect. Microbiol. **13**, 1144210 (2023). 36968107 10.3389/fcimb.2023.1144210 PMC10034137

[82] J. L. Irons, K. Hodge-Hanson, D. M. Downs, RidA proteins protect against metabolic damage by reactive intermediates. Microbiol. Mol. Biol. Rev. **84**, e00024-20 (2020). 32669283 10.1128/MMBR.00024-20 PMC7373157

[83] I. Pecsi , Essentiality of succinate dehydrogenase in *Mycobacterium smegmatis* and its role in the generation of the membrane potential under hypoxia. MBio **5**, e01093-14 (2014). 25118234 10.1128/mBio.01093-14 PMC4145680

[84] T. Hartman , Succinate dehydrogenase is the regulator of respiration in Mycobacterium tuberculosis. PLoS Pathog. **10**, e1004510 (2014). 25412183 10.1371/journal.ppat.1004510 PMC4239112

[85] M. S. Dragset , Genome-wide phenotypic profiling identifies and categorizes genes required for mycobacterial low iron fitness. Sci. Rep. **9**, 11394 (2019). 31388080 10.1038/s41598-019-47905-y PMC6684656

[86] C. Adolph, M. B. McNeil, G. M. Cook, Impaired succinate oxidation prevents growth and influences drug susceptibility in Mycobacterium tuberculosis. MBio **13**, e0167222 (2022). 35856639 10.1128/mbio.01672-22 PMC9426501

[87] R. M. Dedrick , *Mycobacterium abscessus* strain morphotype determines phage susceptibility, the repertoire of therapeutically useful phages, and phage resistance. MBio **12**, e03431-20 (2021). 10.1128/mBio.03431-20 PMC8092298 33785625

[88] R. M. Dedrick , Phage therapy of *Mycobacterium* infections: Compassionate use of phages in 20 patients with drug-resistant mycobacterial disease. Clin. Infect. Dis. **76**, 103–112 (2023). 35676823 10.1093/cid/ciac453 PMC9825826

[89] K. S. Wetzel , Therapeutically useful mycobacteriophages BPs and Muddy require trehalose polyphleates. Nat. Microbiol. **8**, 1717–1731 (2023). 37644325 10.1038/s41564-023-01451-6 PMC10465359

[90] C. L. Dulberger , Mycobacterial nucleoid-associated protein Lsr2 is required for productive mycobacteriophage infection. Nat. Microbiol. **8**, 695–710 (2023). 36823286 10.1038/s41564-023-01333-x PMC10066036

[91] S. A. Binsabaan, K. G. Freeman, G. F. Hatfull, A. P. VanDemark, The cytotoxic mycobacteriophage protein Phaedrus gp82 interacts with and modulates the activity of the host ATPase. MoxR. J. Mol. Biol. **435**, 168261 (2023). 37678706 10.1016/j.jmb.2023.168261 PMC10593117

[92] R. A. Petit, T. D. Read, Bactopia: A flexible pipeline for complete analysis of bacterial genomes. mSystems **5**, e00190-20 (2020). 32753501 10.1128/mSystems.00190-20 PMC7406220

[93] J. E. Barrick , Identifying structural variation in haploid microbial genomes from short-read resequencing data using breseq. BMC Genomics **15**, 1039 (2014). 25432719 10.1186/1471-2164-15-1039 PMC4300727

[94] J. E. Long , Identifying essential genes in *Mycobacterium tuberculosis* by global phenotypic profiling. Methods Mol. Biol. **1279**, 79–95 (2015). 25636614 10.1007/978-1-4939-2398-4_6

[95] New England Biolabs, QC Check and Size Selection using 6% PolyAcrylamide Gel—NEBNext Multiplex Small RNA Sample Prep Set for Illumina (E7300). https://www.neb.com/en-us/protocols/2013/09/09/qc-check-and-size-selection-using-6-polyacrylamide-gel-e7300?srsltid=AfmBOoqUQz-1xYaDcgod_xMJXuiZgyfQs1a_Lvf-UqKdcCbDcWTpAEnR. Accessed 5 October 2020.

[96] A. Stacy, D. Fleming, R. J. Lamont, K. P. Rumbaugh, M. Whiteley, A commensal bacterium promotes virulence of an opportunistic pathogen via cross-respiration. mBio **7**, e00782-16 (2016). 27353758 10.1128/mBio.00782-16 PMC4916382

[97] M. I. Love, W. Huber, S. Anders, Moderated estimation of fold change and dispersion for RNA-seq data with DESeq2. Genome Biol. **15**, 550 (2014). 25516281 10.1186/s13059-014-0550-8 PMC4302049

[98] A. I. Wong, J. M. Rock, CRISPR Interference (CRISPRi) for Targeted Gene Silencing in Mycobacteria. Methods Mol Biol **2314**, 343–364 (2021). 34235662 10.1007/978-1-0716-1460-0_16

[99] M. Richard , Mutations in the MAB_2299c TetR regulator confer cross-resistance to clofazimine and bedaquiline in *Mycobacterium abscessus*. Antimicrob. Agents Chemother. **63**, e01316-18 (2019). 30323043 10.1128/AAC.01316-18 PMC6325171

