# Supplementary material for: The role of colony morphotype in shaping gene essentiality in Mycobacteroides abscessus
Source: Proc Natl Acad Sci U S A. 2025 Sep 30;122(40):e2500719122. doi: 10.1073/pnas.2500719122 (PMC12519085; doi:10.1073/pnas.2500719122)
Supplement: Supplementary file 1 — Appendix 01 (PDF) [file pnas.2500719122.sapp.pdf]

Table S1: SNP differences between MAB^{S} and MAB^{R} ATCC 19977

SNP	Mutation Type	Position in GCF_000069185.1	Mutation	Frequency	Annotation	Gene Locus	Description
1	Missense	1,251,578	A→G	100%	S249G (AGC→GGC)	*MAB_1239* →	Probable PhoH-like protein PhoH2 (phosphate starvation-inducible protein PsiH)
2	Missense	2,623,982	C→G	100%	intergenic (-227/-111)	*MAB_2578c* ← / → *MAB_2579*	hypothetical protein/hypothetical protein
3	Silent	3,187,946	G→A	100%	F155F (TTC→TTT)	*MAB_3147c* ←	Probable conserved polyketide synthase associated protein
4	Missense	3,335,649	G→A	100%	R77H (CGC→CAC)	*MAB_3297* →	Hypothetical protein
5	Missense	3,626,361	A→G	100%	Y278H (TAC→CAC)	*MAB_3579c* ←	Putative acyl-CoA synthase/polyketide synthase
6	Deletion	3,676,120:1	(G)$_{6\to5}$	100%	coding (59/1140 nt)	*MAB_3627c* ←	Possible oxidoreductase
7	Missense	3,712,389	T→A	100%	Y298F (TAC→TTC)	*MAB_3659c* ←	Putative peptidase/amidohydrolase
**8****	**Deletion**	**4,143,843**	**Δ1 bp**	**100%**	**coding (6757/7746 nt)**	***MAB_4098c* ←**	**Non-ribosomal peptide synthase/polyketide synthase**
9	Insertion	4,607,596	+CAT	100%	coding (727/1218 nt)	*MAB_4524* →	hypothetical protein
10	Missense	4,641,134	G→A	100%	S16F (TCC→TTC)	*MAB_4556c* ←	Hypothetical protein
11	Missense	4,837,484	G→A	100%	G390S (GGC→AGC)	*MAB_4724* →	Sodium/calcium exchanger family protein

MAB^{S} reads mapped to MAB^{R} complete genome shown

**Gene mutation known to cause morphotype transition (MABS has a G that was deleted in MABR)

Table S2: Tn-seq samples

Sample Name	Sample Type	Project	Accession	Reads Mapped	Sites (Total)	TA sites occupied (All)	Saturation	Sites (≥0.3 reads/ million)***	TA sites occupied (≥0.3 reads/ million)	Saturation (≥0.3 reads/ million)	Citation
MAB_tnseq_ Smooth_1	Tn-seq	PRJNA1190882	SAMN45060079	12047916	55579	41971	45.84	**40636**	37882	41.38	This work
MAB_tnseq_ Smooth_2	Tn-seq	PRJNA1190882	SAMN45060080	8069749	56876	45154	49.32	**43061**	40327	44.05	This work
MAB_tnseq_ mooth_3	Tn-seq	PRJNA1190882	SAMN45060081	33110781	75171	39838	43.51	**40818**	36848	40.25	This work
MAB_tnseq_ Smooth_4	Tn-seq	PRJNA1190882	SAMN45060082	26101408	73638	38065	41.58	**40005**	35006	38.23	This work
MAB_tnseq_ Rough_1	Tn-seq	PRJNA1190882	SAMN45060083	15710527	77797	58382	63.77	**58280**	55352	60.46	This work
MAB_tnseq_ Rough_2	Tn-seq	PRJNA1190882	SAMN45060084	20550077	74344	56064	61.23	**54004**	51535	56.29	This work
MAB_tnseq_ Rough_3	Tn-seq	PRJNA1190882	SAMN45060085	14037411	85034	54327	59.34	**55204**	50844	55.53	This work
MAB_tnseq_ Rough_4	Tn-seq	PRJNA1190882	SAMN45060086	35861069	89264	52730	57.59	**50926**	46939	51.27	This work
SRR13081049	Tn-seq	PRJNA549322	SAMN16825980	13690537	183323	61363	67.02	61762	54871	59.93	Rifat et al., 2021
SRR13081055	Tn-seq	PRJNA549322	SAMN16825992	5084275	152705	63206	69.04	85429	59869	65.39	Rifat et al., 2021
SRR13081056	Tn-seq	PRJNA549322	SAMN16825991	6432071	211749	61525	67.20	87360	56797	62.04	Rifat et al., 2021
SRR13081057	Tn-seq	PRJNA549322	SAMN16825990	8453235	176581	57561	62.87	98337	53423	58.35	Rifat et al., 2021
SRR13081060	Tn-seq	PRJNA549322	SAMN16825979	8822677	152564	60690	66.29	68071	55990	61.15	Rifat et al., 2021
SRR13081061	Tn-seq	PRJNA549322	SAMN16825978	6891084	161539	62324	68.07	66979	56177	61.36	Rifat et al., 2021
MAB_tnseq_ Smooth_midlog_1	Tn-seq	PRJNA1190882	SAMN45060087	11216658	42894						This work
MAB_tnseq_ Smooth_midlog_2	Tn-seq	PRJNA1190882	SAMN45060088	14097222	42210						This work
MAB_tnseq_ Smooth_midlog_3	Tn-seq	PRJNA1190882	SAMN45060089	9769395	57986						This work
MAB_tnseq_ Smooth_midlog_4	Tn-seq	PRJNA1190882	SAMN45060090	13601281	43266						This work
MAB_tnseq_ Smooth_midlog_5	Tn-seq	PRJNA1190882	SAMN45060091	18680554	46636						This work
MAB_tnseq_ Rough_midlog_1	Tn-seq	PRJNA1190882	SAMN45060092	14835749	43502						This work
MAB_tnseq_ Rough_midlog_2	Tn-seq	PRJNA1190882	SAMN45060093	9055486	36204						This work
MAB_tnseq_ Rough_midlog_3	Tn-seq	PRJNA1190882	SAMN45060094	10336518	51787						This work
MAB_tnseq_ Rough_midlog_4	Tn-seq	PRJNA1190882	SAMN45060095	10351557	45383						This work
MAB_tnseq_ Rough_midlog_5	Tn-seq	PRJNA1190882	SAMN45060096	14326522	47253						This work
MAB_tnseq_ Smooth_abscess_1	Tn-seq	PRJNA1190882	SAMN45060097	8806133	19636						This work
MAB_tnseq_ Smooth_abscess_2	Tn-seq	PRJNA1190882	SAMN45060098	8773136	17880						This work
MAB_tnseq_ Smooth_abscess_3	Tn-seq	PRJNA1190882	SAMN45060099	10024029	24890						This work
MAB_tnseq_ Smooth_abscess_4	Tn-seq	PRJNA1190882	SAMN45060100	7946630	25348						This work
MAB_tnseq_ Smooth_abscess_5	Tn-seq	PRJNA1190882	SAMN45060101	9958864	28803						This work
MAB_tnseq_ Smooth_abscess_6	Tn-seq	PRJNA1190882	SAMN45060102	2311110	9904						This work
MAB_tnseq_ Smooth_abscess_7	Tn-seq	PRJNA1190882	SAMN45060103	1667188	20099						This work
MAB_tnseq_ Smooth_abscess_8	Tn-seq	PRJNA1190882	SAMN45060104	8169790	21618						This work

MAB_tnseq_ Smooth_abscess_9	Tn-seq	PRJNA1190882	SAMN45060105	7042104	26053						This work
MAB_tnseq_ Rough_abscess_1	Tn-seq	PRJNA1190882	SAMN45060106	3433254	11820						This work
MAB_tnseq_ Rough_abscess_2	Tn-seq	PRJNA1190882	SAMN45060107	8350466	12132						This work
MAB_tnseq_ Rough_abscess_3	Tn-seq	PRJNA1190882	SAMN45060108	7804317	10589						This work
MAB_tnseq_ Rough_abscess_4	Tn-seq	PRJNA1190882	SAMN45060109	10034261	18069						This work
MAB_tnseq_ Rough_abscess_5	Tn-seq	PRJNA1190882	SAMN45060110	9374869	15488						This work
MAB_tnseq_ Rough_abscess_6	Tn-seq	PRJNA1190882	SAMN45060111	8066108	14314						This work
MAB_tnseq_ Rough_abscess_7	Tn-seq	PRJNA1190882	SAMN45060112	14154545	25078						This work
MAB_tnseq_ Rough_abscess_8	Tn-seq	PRJNA1190882	SAMN45060113	7846455	19420						This work
MAB_tnseq_ Rough_abscess_9	Tn-seq	PRJNA1190882	SAMN45060114	20419276	53081						This work
MAB_tnseq_ Rough_abscess_10	Tn-seq	PRJNA1190882	SAMN45060115	6637146	43606						This work
MAB_tnseq_ Rough_abscess_11	Tn-seq	PRJNA1190882	SAMN45060116	5321125	16353						This work
MAB_tnseq_ Smooth_RoughHK_ abscess_1	Tn-seq	PRJNA1190882	SAMN45060117	6999608	20350						This work
MAB_tnseq_ Smooth_RoughHK_ abscess_2	Tn-seq	PRJNA1190882	SAMN45060118	11077661	28203						This work
MAB_tnseq_ Smooth_RoughHK_ abscess_3	Tn-seq	PRJNA1190882	SAMN45060119	5801894	22603						This work
MAB_tnseq_ Smooth_RoughHK_ abscess_4	Tn-seq	PRJNA1190882	SAMN45060120	10156073	24784						This work
MAB_tnseq_ Smooth_RoughHK_ abscess_5	Tn-seq	PRJNA1190882	SAMN45060121	4177647	21373						This work
MAB_tnseq_ Smooth_RoughHK_ abscess_6	Tn-seq	PRJNA1190882	SAMN45060122	10417695	21148						This work

*** The site data described in the text is in bold. Notice that the raw site count may be higher than the possible number of TA sites, 91556. We chose to count sites with >= 0.3 reads per million because that equates to > sites being true TA sites.

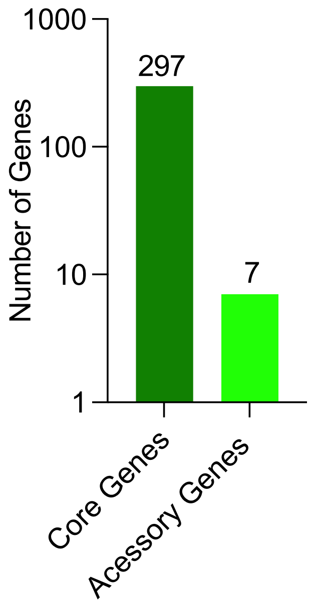

Fig. S1. 298 of the 304 conserved MAB essential genes are in the core genome. Core genes are defined as being present in 95% of 34 globally representative MAB genomes**.** The six essential genes not in the core genome are MAB_0210, MAB_0222c, MAB_0441, MAB_1556, MAB_4318, MAB_4475, MAB_4828c (**Table S4**).

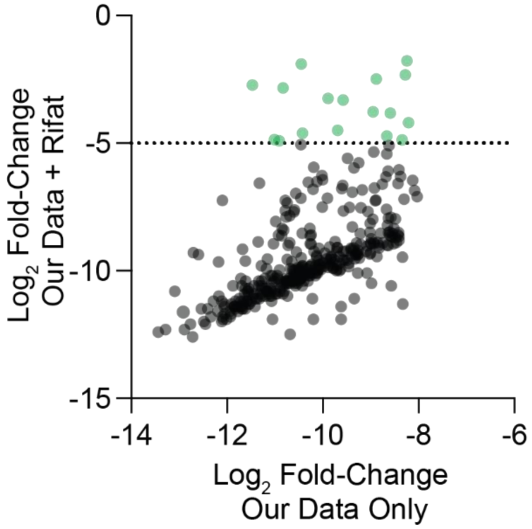

Fig. S2. Removing previously published Tn-seq data from our analysis has minimal impact on MAB essential genes. To test the robustness of our dataset, we compared the list of essential genes with and without inclusion of previously published data from Rifat *et al*. 261 of the 304 essential genes found using all datasets were identified as essential using only the data generated in this study. While not classified as 'essential', 17 of the 43 genes have a $\log_2$-fold change of ≤ 5, indicating that they have fitness defects.

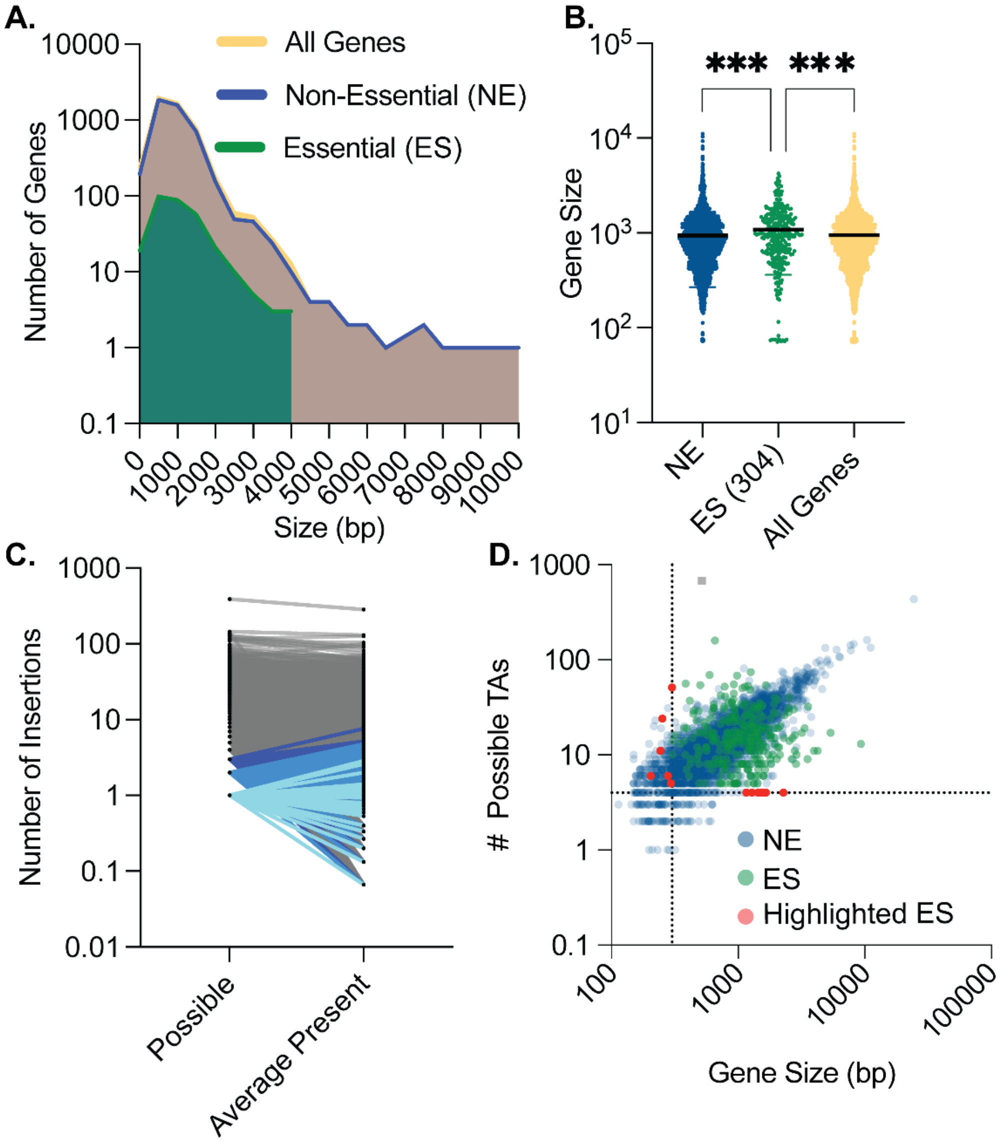

Fig. S3. Essential genes are not biased toward small genes, and several small genes have transposon insertions. (A-B) Gene size distribution of essential (ES), non-essential genes (NE), and all genes. *** p≤0.001 using a Kruskal Wallis test. **(C)** The possible TA sites per gene plotted along with the average number of insertions present in the transposon libraries. Genes with 1 possible insertion site (TA) are in light blue (—), genes with 2 in medium blue (—), genes with 3 possible sites in dark blue (—). All other genes are in gray. **(D)** The average number of TA sites containing a transposon is plotted against gene size. Six genes smaller than 300 bp and 9 genes with 4 TA sites, were found to be essential (*P-value* ≤-6.97 x10^{-35}). Non-essential genes are in blue (●), essential genes are in green (●), and essential genes that are ≤ 300base pairs or ≤ 4 possible TA sites are in red (●).

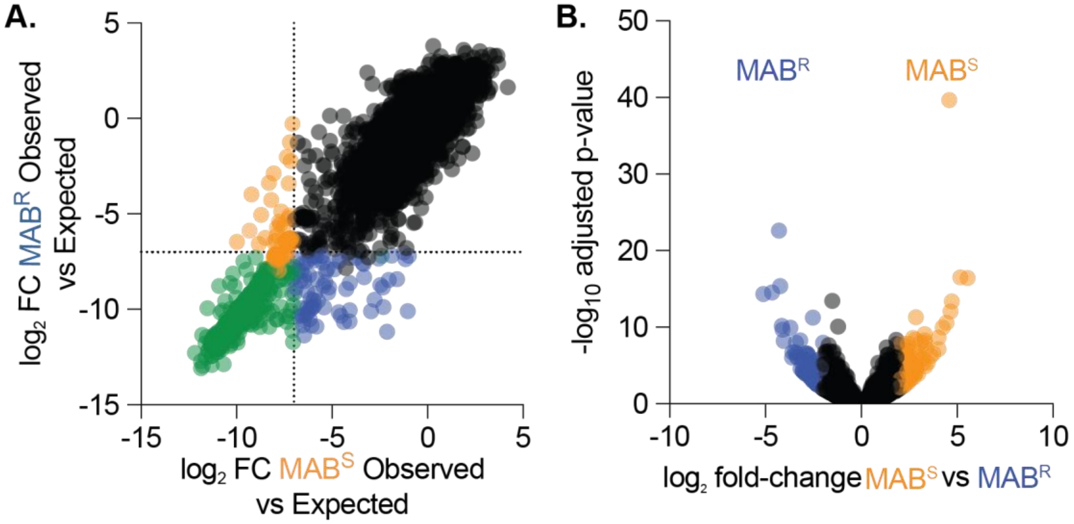

Fig. S4. Identification of essential genes using Monte Carlo and differential transposon frequency analysis. (A) Identification of MAB^S and MAB^R essential genes using Monte Carlo analysis. Shown are the log_2-transformed differences between observed and fitness-neutral in silico generated transposon frequencies (pseudodata) for each gene (circles) for both morphotypes. **(B)** Identification of MAB^S and MAB^R genes important for fitness using differential transposon frequency analysis. The shared genes between these two analyses are shown in Fig. 2A. (●) are MAB^R-specific essential genes, (●) are MAB^S-specific essential genes, and (●) are shared essential genes.

A.

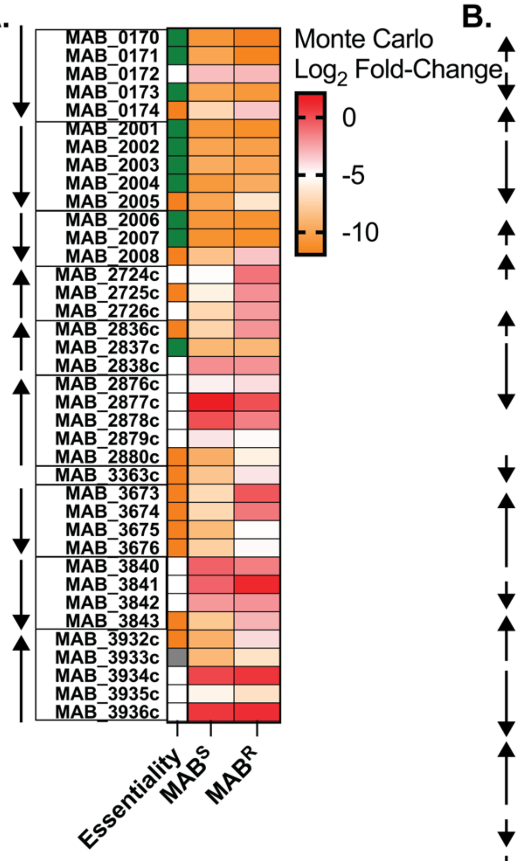

B.

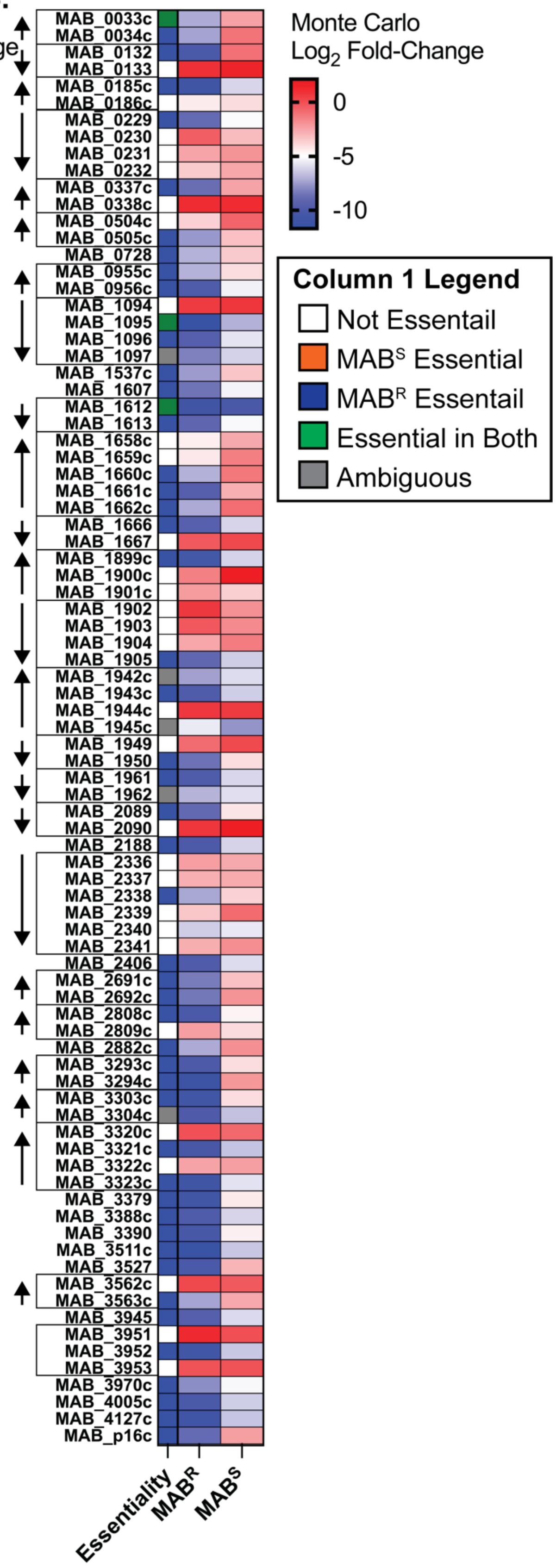

Fig. S5. Polar effects of transposition do not explain gene essentiality differences between morphotypes. (A) MAB^S and **(B)** MAB^R essential genes are displayed in their predicted operons with orientation depicted by an arrow. 13/60 essential genes are in an operon with another morphotype-specific essential gene. 7/60 genes are in an operon with a gene essential in both morphotypes. 4/60 genes (called ambiguous) are essential by Monte Carlo analysis but not by differential transposon frequency.

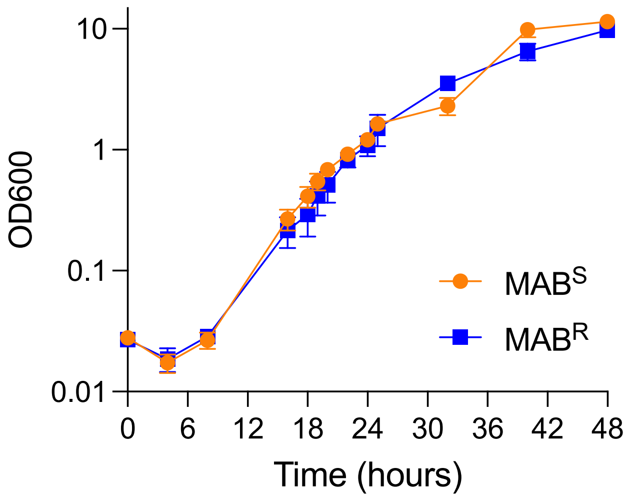

Fig. S6. MAB morphotypes grow at the same rate. Growth curves of MAB^S and MAB^R grown in the same conditions as those used for Tn-seq out-growth. The doubling times from 3 independent experiments in biological triplicate were not statistically different ($MAB^S = 4.92 \pm 0.49$ hours and $MAB^R = 4.93 \pm 0.64$ hours). Representative growth curves are shown.

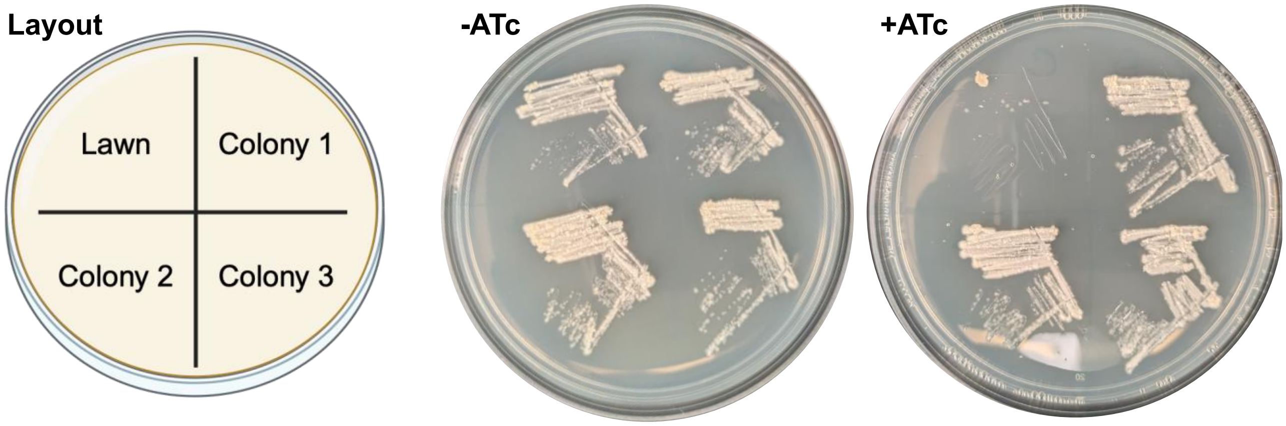

Fig. S7. sdh2C CRISPRi resistant colonies are stable. MAB^R colonies (Colony 1, 2, and 3) that grew in the *sdh2C* CRISPRi zone of inhibition (**Fig. 3A**) were restreaked on Km plates with or without the ATc inducer. As a control, bacteria from the bacterial lawn outside the zone of inhibition were also restreaked (Lawn).

Table S3: SNP Analysis Results Following Sequencing of SdhC (MAB_3673) CRISPRi ATc Resistant and Control Colonies

**ATc Resistant Colony 1**					
**Position in MAB**	**Mutation**	**Frequency**	**Annotation**	**Gene**	**Description**
520,324	C→T	93.10%	intergenic (-225/-42)	*MAB_1508c* ← / → MAB_1509	42 bp upstream of MAB_1509 alpha/beta-hydrolase family protein/hypothetical protein
**Position in Integrated Plasmid**	**Mutation**	**Frequency**	**Annotation**	**Gene**	**Description**
169	214 bp→24 bp	100%	intergenic (–/–)	– / –	–/– (downstream of sgRNA)

**ATc Resistant Colony 2**					
**Position in MAB**	**Mutation**	**Frequency**	**Annotation**	**Gene**	**Description**
441,994	G→A	93.20%	intergenic (-95/+32)	*MAB1570* ← / ← *MAB_1569*	95 bp upstream of MAB_1570 hypothetical protein
**Position in Integrated Plasmid**	**Mutation**	**Frequency**	**Annotation**	**Gene**	**Description**
169	214 bp→24 bp	100%	intergenic (–/–)	– / –	–/– (downstream of sgRNA)

**ATc Resistant Colony 3**					
**Position in MAB**	**Mutation**	**Frequency**	**Annotation**	**Gene**	**Description**
520,324	C→T	70.20%	intergenic (-225/-42)	*MAB_1508c* ← / → MAB_1509	42 bp upstream of MAB_1509 alpha/beta-hydrolase family protein/hypothetical protein
2,537,133	G→C	79.20%	R177G (CGC→GGC)	*MAB_4493* ←	mutation of MAB_4493 IclR family transcriptional regulator
**Position in Integrated Plasmid**	**Mutation**	**Frequency**	**Annotation**	**Gene**	**Description**
169	214 bp→24 bp	100%	intergenic (–/–)	– / –	–/– (downstream of sgRNA)

**Control Colony**					
**Position in MAB**	**Mutation**	**Frequency**	**Annotation**	**Gene**	**Description**
None					
**Position in Integrated Plasmid**	**Mutation**	**Frequency**	**Annotation**	**Gene**	**Description**
169	G→T	100%	intergenic (–/–)	– / –	–/– (downstream of sgRNA)
171	2 bp→TC	100%	intergenic (–/–)	– / –	–/– (downstream of sgRNA)
174	G→A	100%	intergenic (–/–)	– / –	–/– (downstream of sgRNA)
176	4 bp→GACG	100%	intergenic (–/–)	– / –	–/– (downstream of sgRNA)
181:01:00	+GA	100%	intergenic (–/–)	– / –	–/– (downstream of sgRNA)
183	5 bp→5 bp	100%	intergenic (–/–)	– / –	–/– (downstream of sgRNA)
274	$(T)_{10\rightarrow 9}$	100%	intergenic (–/–)	– / –	–/– (downstream of sgRNA)

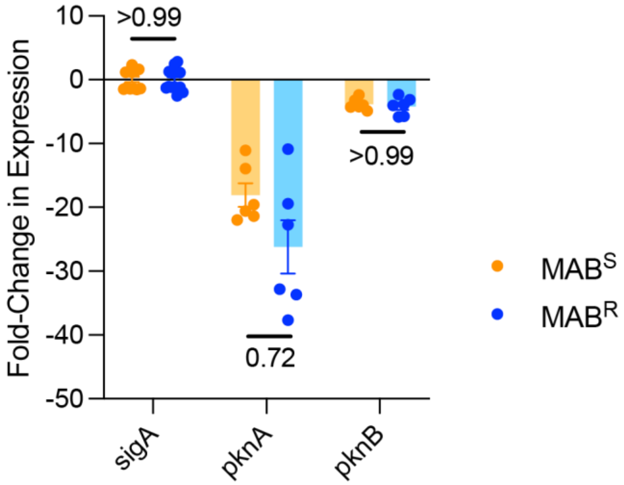

Fig. S8. CRISPRi targeting *pknA* reduces mRNA levels similarly in both MAB morphotypes. MAB^S and MAB^R carrying the Cas9 plasmid with an sgRNA targeting *pknA* were grown for 16 hours in the presence of the sgRNA inducer (ATc). Total RNA was isolated and mRNA levels of *pknA* and *pknB* were quantified using q*R*T-qPCR after normalization to *sigA* mRNA levels. Data were analyzed using the Mann-Whitney test, and p-values are shown. Targeting of *pknA* resulted in an average decrease in mRNA of 18- and 26-fold in MAB^S and MAB^R, respectively. Expression of the downstream gene *pknB* was reduced by ~4-fold in both morphotypes.

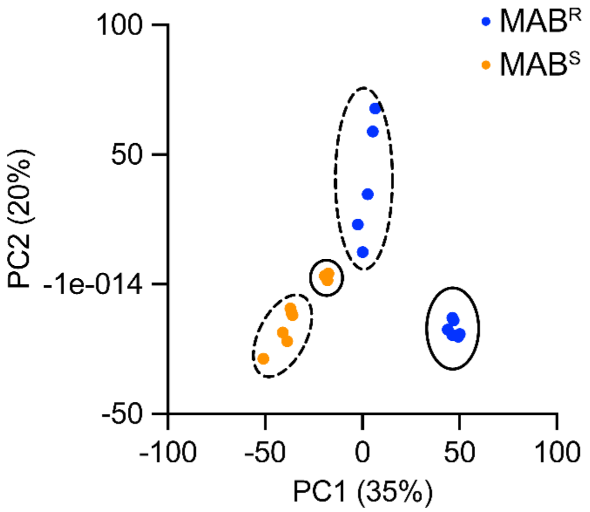

Fig. S9. Tn-seq abscesses samples cluster by morphotype. Abscess Tn-seq samples (circles) were normalized by read depth per gene and variance stabilized transformed (blind = TRUE) prior to Principal Component Analysis. Manhattan clustering revealed 4 clusters denoted by the circles. The dashed and solid circles represent the biological replicates with each morphotype. MAB^{S} abscess infections are in orange (•) and MAB^{R} infections in blue (•).

Table S4: Strains used in this work

Strain	Description	Origin
MABS ATCC 19977	Isolated from reconstituted ATCC lyophilized stock	This work
MABR ATCC 19977	Isolated from reconstituted ATCC lyophilized stock	This work
MABS pILR2	Vector Control	This work
MABR pILR2	Vector Control	This work
MABS pILR2::rpoB	repression of rpoB	This work
MABR pILR2::rpoB	repression of rpoB	This work
MABS pILR2::MAB_0033i	repression of pknB	This work
MABR pILR2::MAB_0033i	repression of pknB	This work
MABS pILR2::MAB_3673i	repression of sdh2C	This work
MABR pILR2::MAB_3673i	repression of sdh2C	This work
MABS pILR2::MAB_3674i	repression of sdh2D	This work
MABR pILR2::MAB_3674i	repression of sdh2D	This work
MABS pILR2::MAB_3675i	repression of sdh2A	This work
MABR pILR2::MAB_3675i	repression of sdh2A	This work
MABS pILR2::MAB_3676i	repression of sdh2B	This work
MABR pILR2::MAB_3676i	repression of sdh2B	This work
MABS pILR2::MAB_3363i	repression of FixA	This work
MABR pILR2::MAB_3363i	repression of FixA	This work
MABS pILR2::MAB_0174i	repression of MAB_0174	This work
MABR pILR2::MAB_0174i	repression of MAB_0174	This work
MABS pILR2::MAB_0034i	repression of pknA	This work
MABR pILR2::MAB_0034i	repression of pknA	This work
MABS pILR2::MAB_2089i	repression of MAB_2089	This work
MABR pILR2::MAB_2089i	repression of MAB_2089	This work
MABS pILR2::MAB_2338i	repression of argB	This work
MABR pILR2::MAB_2338i	repression of argB	This work
MABS pILR2::MAB_3536i	repression of MAB_3536	This work
MABR pILR2::MAB_3536i	repression of MAB_3536	This work
EN38 MABS pILR2::MAB_0034i	repression of pknA in a smooth clinical isolate	This work
EN40 MABR pILR2::MAB_0034i	repression of pknA in a rough clinical isolate	This work
EN55 MABS pILR2::MAB_0034i	repression of pknA in a smooth clinical isolate	This work
EN58 MABR pILR2::MAB_0034i	repression of pknA in a rough clinical isolate	This work
MABSΔpknA	clean isogenic pknA deletion mutant generated using pUX1-katG	This work
MABSΔMAB_3590c:zeoR	MAB_3590c mutated and replaced with a zeocin resistance cassette using pJV53	This work
MABSΔMAB_2606c:zeoR	MAB_2606c mutated and replaced with a zeocin resistance cassette using pJV53	This work

Table S5: Primers used in this work

Name	Sequence	Use
Tn-seq primers (not specific to MAB)	see Tn-Seq Illumina Library Preparation from Initial PA14 Mutant Library in supplemental material for ref. (43)	Existing Tn-seq workflow
Tn-1 MycoMarTspn	/5Biosg/GC CTT CTT GAC GAG TTC TTC TGA GCG	MAB Specific Tn-seq primers
Tn-2 MycoMar	AATGATACGGCGACCACCGAGATCTACACTCTTTC CCTACACGACGCTCTTCCGATCTNNNNNN CGGGGACTTATCAGCCAACC	MAB Specific Tn-seq primers
cr.23a (*rpoB*)	GGGAGAACGACAGCGACATCGAGCC	CRISPRi Akusobi *et al*., 2022
cr.23b (rpoB)	AAACGGCTCGATGTCGCTGTCGTTC	CRISPRi Akusobi *et al*., 2022
MAB_3673iF	GGGAGTATCCAAGACGTGGACAAAGAG	CRISPRi
MAB_3673iR	AAACCTCTTTGTCCACGTCTTGGATAC	CRISPRi
MAB_3674iF	GGGAGAACGCTCGTGTGCGAACCGGTC	CRISPRi
MAB_3674iR	AAACGACCGGTTCGCACACGAGCGTTC	CRISPRi
MAB_3675iF	GGGAGCGTACCGATGTTCCTGAATGATG	CRISPRi
MAB_3675iR	AAACCATCATTCAGGAACATCGGTACGC	CRISPRi
MAB_3676iF	GGGAGCGGTCCGTGGGCAGGCAGGGTAC	CRISPRi
MAB_3676iR	AAACGTACCCTGCCTGCCCACGGACCGC	CRISPRi
MAB_3363i	GGGAGCCCAGGTACTCGGCGATGATCG	CRISPRi
MAB_3363i	AAACCGATCATCGCCGAGTACCTGGGC	CRISPRi
MAB_0174i	GGGAAACCATCACGGCCGCACGCCCGG	CRISPRi
MAB_0174i	AAACCCGGGCGTGCGGCCGTGATGGTT	CRISPRi
MAB_0034i	GGGAGCGCTCGATGAATTCGGGGTCCGA	CRISPRi
MAB_0034i	AAACTCGGACCCCGAATTCATCGAGCGC	CRISPRi
MAB_2089i	GGGAACGCCTGCGTCGACCAAGCGGG	CRISPRi
MAB_2089i	AAACCCCGCTTGGTCGACGCAGGCGT	CRISPRi
MAB_2338i	GGGAGACCGGGTGGATACCACAGTTGC	CRISPRi
MAB_2338i	AAACGCAACTGTGGTATCCACCCGGTC	CRISPRi
MAB_3536i	GGGAATGCCGTGGTGGCCGGTCCAGGT	CRISPRi
MAB_#536i	AAACACCTGGACCGGCCACCACGGCAT	CRISPRi
MAB_0034 F1F	GAGATTAATTAA agattctcgcgatggtctcca	pknA Mutagenesis in MABS
MAB_0034 F1R	GAGACAATTGtcatgaggccccctgtagtgc	pknA Mutagenesis in MABS
MAB_0034 F2F	Atgaccactccgcagcacctc	pknA Mutagenesis in MABS
MAB_0034 F2R	GAGAGCTAGCctagcccgcgccgaagcgcag	pknA Mutagenesis in MABS
sigA_3009 FWD	CCAGGTTGCCTTCCTGAAT	RT-qPCR
sigA_3009 REV	CCAAGAACCATCTGCTGGAA	RT-qPCR
PknA_34 FWD	TCTACTCACTGGGAGTGGTT	RT-qPCR
PknA_34 REV	GGCGTTTCCTTGATGTGTTTC	RT-qPCR
pknB 33 FWD	CCGAAGTCCATCACCTTCAC	RT-qPCR
pknB 33 REV	CACTCAACTTCAGCCACCA	RT-qPCR
